# Electric vehicle charging stations: Model, algorithm, simulation, location, and capacity planning

**DOI:** 10.1016/j.heliyon.2024.e29153

**Published:** 2024-04-06

**Authors:** Serdar Çelik, Şeyda Ok

**Affiliations:** a Department of Management Information Systems, Ostim Technical University, Ankara, Turkey; b Department of Marketing, Ostim Technical University, Ankara, Turkey

**Keywords:** Electric vehicle, Charging station, Genetic algorithm, Simulation, P-median, Facility location

## Abstract

The transition to sustainable transportation is imperative in mitigating environmental impacts, with electric vehicles (EVs) at the forefront of this shift. Despite their environmental benefits, the global adoption of EVs is curtailed by challenges such as nascent battery technology, high costs, and insufficient charging infrastructure. This study addresses the optimizing electric vehicle charging station (EVCS) locations as a critical step toward enhancing EV adoption rates. Thus, establishing efficient charging stations is critical to meet the increasing demand. By integrating location modeling with demand forecasts and market penetration, we propose a comprehensive approach to determine optimal locations and capacities for EVCS. Firstly, review existing literature, highlighting the significance of facility location models in optimizing EV charging infrastructure and identifying gaps in addressing demand and market penetration. Our methodology uses a genetic algorithm to solve the p-median problem for location selection and Arena 14 simulation software to model station traffic and optimize charging unit types and quantities. The model prioritizes public areas, considering potential demand points and station locations to propose optimal charging areas. Results indicate that our model minimizes travel distances and waiting times, offering a scalable solution adaptable to future EV market growth. This study contributes to the field by presenting a sustainable and economical model for EVCS placement and capacity planning, underlining the importance of a robust charging network in the broader adoption of electric transportation. The findings suggest that proactive infrastructure development, guided by accurate demand predictions and optimized location strategies, can significantly enhance the feasibility and attractiveness of EVs, supporting global efforts towards a cleaner, more sustainable transportation system.

## 1 Introduction

In the quest for sustainable transportation solutions, EVs have emerged as a promising alternative to internal combustion engine vehicles, offering significant potential in reducing greenhouse gas emissions and dependence on oil. The widespread adoption of EVs not only promises a substantial decrease in global fossil fuel consumption but also presents the possibility of operating a much cleaner road transport system, virtually free from exhaust emissions [1]. Considering the transportation sector, one of the significant contributors to greenhouse gas emissions, adopting energy-efficient and environmentally friendly EVs can directly reduce fuel dependency and has undoubtedly become a priority target for optimization [2]. The European Union, recognizing the urgency of reducing greenhouse gas emissions originating from the transportation sector, which constitutes a significant portion of total energy consumption, has highlighted the development of EVs as a corrective measure [3]. On the other hand, the growing public awareness of environmental protection also accelerates the strong growth of EVs [[4], [5], [6]]. However, despite their advantages and increasing support worldwide, the global market penetration of EVs remains relatively low, hindered by challenges such as immature battery technology, high initial costs, and inadequate charging infrastructure [7,8].

The widespread adoption of EVs is intrinsically linked to the presence of a robust charging infrastructure, necessitating the joint efforts of countries, regions, and municipalities. Establishing such an infrastructure is often cited as a critical factor in increasing the adoption rates of EVs. As advancements in battery technology prepare to expand the usage range of EVs further, there is a parallel need for the expansion of electric charging networks. This expansion highlights the importance of developing the electric grid infrastructure and underlines the need to evaluate and adapt station capacities. To overcome these challenges, location modeling for charging stations has become a significant area of research, aiming to predict demand and optimize the placement of charging facilities. Various methodologies, including optimization algorithms such as genetic algorithms, integer programming, and geographical and statistical approaches, have been utilized to determine the most suitable locations for charging stations [[9], [10], [11]]. These studies aim to minimize costs and reduce journey lengths and consider the spatial distribution of charging demand based on available data and simulated scenarios.

By 2030, EVs are expected to reach 120 million [12,13]. With the rapid development of EVs, the demand for charging infrastructures, which are critical for their practical use, is anticipated to increase. The integration of EVs into transportation systems and the consequent development of charging infrastructure bring along complex issues that require innovative solutions. Numerous studies have been conducted on the placement of charging stations to meet the increasing demand for charging. Previous models for the placement of EVCSs have often been based on the assumption of a fixed charging demand and have not sufficiently considered the number of EVs introduced to the market. This situation is likely to inaccurately reflect the charging habits of electric vehicle users, leading to the incorrect placement of stations.

In response, this study first focuses on the demand-driven locations of EVCS. Then, it determines station capacities by considering the types of charging units at the station relative to the number of EVs entering the market. The work introduces a sustainable and economical model for selecting facility locations and determining their capacities. This model focuses on selecting public areas, considering demand points and potential charging station locations to determine optimal charging station areas. Addressing the complexity of facility location challenges, the research introduces a mathematical model based on the p-median problem to determine charging station locations without initial capacity assessments. The facility location model, determined using a genetic algorithm, is followed by simulation techniques to determine station capacities and the types and quantities of charging units at each station. Based on simulation data, the study evaluates station traffic density and develops various scenarios using Arena 14 software to optimize the types and quantities of charging units to alleviate waiting times.

**Fig. 1:**
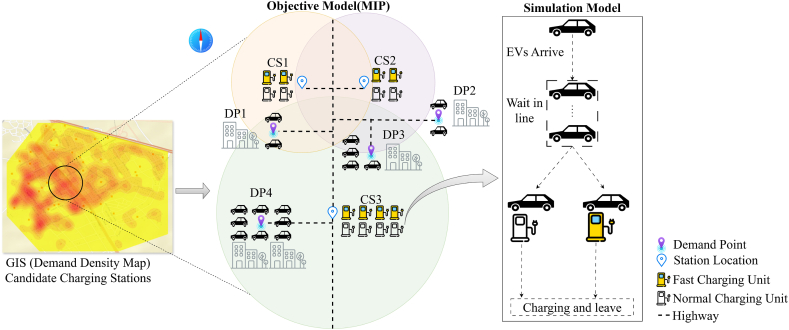
The main structure of EVCSs location and capacity planning.

Fig. 1 illustrates the charging process of an EVs. In Fig. 1., there are locations where EVs are parked and candidate charging station points. CS1, CS2, and CS3 are charging stations. CS1 is close to CS2. The circle represents the service interval of the charging station. When there is a charging request, the driver arrives at the nearest charging station and performs fast or normal charging. If the EVs at demand points DP1, DP2, DP3, and DP4 need to be charged, they can choose to go to charging stations CS1, CS2, or CS3. If the EV at DP1 needs to be charged, it has to choose between CS1 and CS3 and go to the nearest charging station. We see that the CS1 station is more reasonable and objective. Since CS1, CS2, and CS3 are close to each other, the intersection of the orange, purple, and green circle in the figure is rich in charging resources and meets the charging demand of this area to a large extent. However, the uneven distribution of charging sources brings some problems, such as difficulty in charging for users. If one wants to charge an electric vehicle at DP2, it turns out that it is not suitable for charging the electric vehicle. In order to correct this illogical situation, in this study, when an electric vehicle needs to be charged, its current location is considered as a potential charging demand point, and a more suitable location is determined among the candidate stations. The study also focuses on determining the number of fast and normal charging station units to avoid queuing inside the station once the stations are identified. The contributions of this paper are as follows.

1) At present, most of the research on the location of charging stations does not fully understand the charging demand of each station, and it is difficult to meet the interests of all parties. For this reason, this article eliminates the waiting time of users at the station by creating the location model in public areas and determining the capacities of the stations and the type of charging units in these stations with a simulation approach.
2) A prediction model of electric vehicle charging demand was created based on parking data of EV users. Taking into account electric vehicle charging times, the number and type of units required to eliminate queue waiting times within the station were determined by scenarios.
3) A location selection model targeting minimum transportation cost has been established. Public areas were selected to install candidate charging stations.

The article is organized as follows. Section 2 introduces the literature and shows which gaps in the literature the study fills. Section 3 describes the development of the charging station location optimization model and genetic algorithm. Section 4 describes the solution method and simulation method of the location model; and Section 5 is the summary of the study.

## 2 Literature review

The optimization of EVCS locations constitutes a paramount area of inquiry within the academic domain, with various facility location models being deployed to address this intricate issue [[14], [15], [16]]. Facility location optimization is fundamentally regarded as an investment strategy poised to yield substantial economic returns, necessitating a strategic balance between maximizing benefits and minimizing operational expenditures within a set of predefined constraints. Extensive scholarly efforts have been dedicated to the refinement of facility location models, incorporating a range of constraints tailored to the specificities of the problem at hand or omitting those deemed extraneous. Despite these advancements, the optimization of EV charging infrastructure continues to encounter several challenges. Notably, an increase in the driving range post-charging may render the initial station locations suboptimal, implicating a need for additional facilities to maintain service quality and necessitating ongoing reevaluation to accommodate market dynamics and enhanced penetration rates. A further complicating factor lies in the diversity of optimization models applicable to facility configuration, with variations in model selection and solution strategies significantly impacting the alignment of theoretical outcomes with practical realities. Consequently, each model demands careful consideration of factors such as electric transmission costs, charging station congestion, and technological evolutions, including enhancements in battery technology that extend vehicle range.

In light of these considerations, the literature offers extensive discourse on general location theory, including the exploration of the p-median problem, to inform the development of more effective charging infrastructure. This study undertakes a comprehensive literature review to distill the core principles underlying various optimization approaches and delineate the unique contributions of the present investigation relative to existing scholarship. The focal point of this analysis encompasses three predominant models widely recognized and utilized within scholarly circles for addressing EVCS location challenges: the p-median problem, the set covering problem, and the fixed charge problem, as shown in Table 1. Each model is detailed, highlighting its applicability and potential for addressing the complexities associated with EVCS deployment. Additionally, the relevance of Origin-Destination (O-D) trip-based models is examined, underscoring their suitability for capturing the nuanced dynamics of realistic scenarios. This exploration encompasses a thorough review of pertinent studies, providing a foundational understanding of each model's methodological underpinnings and practical implications for facility location optimization in the context of EV charging infrastructure.

**Table 1:** Classification of EVCSLP papers according to solution approaches and implications.

Ref.	Model			Algorithm	Simulation	Station Location	Charger Type	Demand	Implications
	Type	Objective Function							
[23]	–	$\mathrm{Min}\sum_{t=1}^{T}\frac{1}{{\left(1+n\right)}^{t}}\left[\sum_{i=1}^{N_{EVCS}}(C_{EVCS}^{I}\left(t\right)+(C_{EVCS}^{O}\left(t\right)+(C_{EVCS}^{M}\left(t\right)+(C_{PS}^{L}\left(t\right)\right]$	Total cost	MPDIPA	Iteration	Node	Voltage limit	Daily journey	The developed model significantly decreases the duration of the procedure.
[24]	MINLP	$\mathrm{Min}\sum_{i=1}^{NC}\left({DC}_{i}+{EC}_{i}\right)X_{i}+\sum_{j=1}^{NEV}{EVL}_{j}+\sum_{n=1}^{NB}{GLC}_{n}$	Total cost	GA	–	Public	EVFC	500 EV assumed	Electric grid losses constitute a significant portion of the overall cost.
[25]	MIP	$\mathrm{Min}\;\sum_{i}\sum_{j}c_{ij}y_{ij}$	Total access cost	–	–	Public	EVNC	Parking time	The developed model can be applied in other cities and regions as well.
[30]	MIP	$\mathrm{Max}\sum_{p\in P}F^{p}w^{p},\mathrm{Min}\sum_{i\in N}\sum_{k\in K}C_{i}^{k}X_{i}^{k}$	Coverage of EV flows	–	–	Node	EVNC	EV routing behavior	The developed models use a single objective for maximizing the coverage.
[32]	LP	$\mathrm{Min}\left\{F=\sum_{t=1}^{T}\sum_{i=1}^{I}\sum_{k=1}^{K}\frac{1}{w_{i}}c_{i,k}x_{i,t}\right\}$	Total cost	–	–	Node	EVNC EVFC	User behavior	Higher single-use charging time lowers the overall development cost of EV charging stations.
[33]	IP	$\mathrm{Max}\;\sum_{q\epsilon Q}\lambda_{q}{\bar{X}}_{q}$	Demand coverage	–	–	Node	EVFC	User behavior	Service time is a stochastic factor.
[34]	CMC	$\mathrm{Max}\sum_{i\in I}\sum_{t\epsilon T}d_{it}^{L2}\sum_{j\epsilon J}z_{itj}^{L2}+\sum_{i\in I}\sum_{t\epsilon T}d_{it}^{L3}\sum_{j\epsilon J}z_{itj}^{L3}$	Total service time	–	ABS (MATsim)	Public	EVNC EVFC	Daily journey	The model showed a consistent relationship between estimated charging demand and actual energy consumption.
[35]	IP	$\mathrm{Max}\sum_{1}^{n}s_{i}x_{i}$	Demand coverage	–	–	Public	EVNC	User behavior	EV penetration rate gives the value of energy demand.
[36]	MIP	$\mathrm{Min}\;\sum_{i\in I}\sum_{j\in N}a_{ij}{h_{i}U}_{ij}$	Distance coverage	–	–	Public	EVNC	Parking behaviors	It is recommended to use the proposed coverage model in industrial zones, cities and microgrids.
[37]	MCLP	$\mathrm{Max}\;\sum_{i\in I}a_{i}y_{i}$	Demand coverage	–	–	Node	EVFC	User behavior	Placing fast chargers at petrol stations increases demand coverage by over 50% versus current fast charging locations.
[51]	SP	$\mathrm{Min}\sum_{i\in F}f_{i}x_{i}+\sum_{s\epsilon S}p_{s}\sum_{j\epsilon D}\sum_{i\epsilon F}d_{j}c_{ij}y_{ij}^{s}$	Total fixed cost	PSO	Iteration	Node	–	Sampled	These results show that the PSO is able to improve the solution quality noticeably.
[52]	MIP	$\mathrm{Min}\sum_{i=1}^{n}\sum_{j=1}^{m}c_{ij}x_{ij}+\sum_{i=1}^{n}f_{i}y_{i}$	Total fixed cost	–	–	Node	EVNC EVFC	Daily journey	Demonstrates the applicability of the modeling approach for hybrid EVs.
[53]	NLP	${\mathrm{Max}}_{x,r}\sum_{j\in J}P_{j}=\sum_{j\in J}\left(U_{j}-C_{j}\right)$	Total profit	GA	Iteration	Network	EVFC	Elastic	The significance of aligning the power grid capacity with the level of charging demand becomes evident.
[54]	IP	$\mathrm{Min}\;\sum_{i}\sum_{j}c_{ij}X_{ij}$	Total cost	BB	–	Node	BES	–	The model identifies the best locations and quantity of stations by factoring in battery range, routes, ES fleet size, location and service capacities, and costs.
[57]	–	–	Performance of EV	–	DES (Flexim)	Node	EVNC	Random	Exceeding station capacity lowers service quality.
[60]	MILP	${\mathrm{Max}}_{x_{k}y_{r}^{w}}\sum_{w\in W}\sum_{r\in R^{w}}X_{r}^{w}\varphi_{r}^{w}$	Path flow	–	–	Network	EVFC	Travel time	Driving range significantly impacts optimal charging station placement.
[61]	–	$\mathrm{Min}\;F=F_{C1}+F_{C2}+F_{C3}$	Operation, time, penalty cost	WOA	MCS	Node	EVFC	Dynamic	The optimum number of charging stations indicates that the comprehensive cost is lowest, and the energy saving and emission reduction effect is good.
[62]	MILP	$\mathrm{Min}\sum_{\left(i,j\right)\in A}x_{ij}c_{ij}+\sum_{\left(r,s\right)\in W}p_{rs}q_{u}^{rs}$	Total cost	BB	–	Node	EVNC	Dynamic	More charging stations result in lower travel costs.
[63]	MILP	$\mathrm{Min}\;\sum_{t=1}^{T}\left[\delta_{t}^{inv}c_{t}^{inv}\left(x_{t},P_{t}{,P}_{t-1}\right)+\delta_{t}^{oper}c_{t}^{oper}(r_{t}y_{t}P_{t}\right]$	Total cost	–	Iteration	Node	EVNC	User behavior	The method lowers grid costs for EV charging needs.
[64]	MIQP	$\mathrm{Min}\;C\left(N^{ch}{,y}^{cs},l,D^{tp}\right)=c^{inv}\left(N^{ch},y^{cs}\right)+c^{oper}\left(l,D^{tp}\right)$	Investment cost	FWA	Iteration	Node	Power rating	User behavior	The method can effectively reduce the investment.
[66]	–	$\mathrm{Min}\;F_{cost}=C_{i}^{c}+C_{i}^{r}+C_{i}^{d}$	Total cost	PSO	–	Node	EVNC	User behavior	Geographical data significantly improves construction cost estimations in the model.
[70]	IP	$MinJ\left(x,y\right)=C\sum_{j=1}^{N}x_{j}+V\sum_{j=1}^{N}y_{j}$	Investment cost	–	–	Node	EVFC	User behavior	The taxi fleet's residence pattern determines the location of charging stations.
[71]	SP	${\mathrm{Min}}_{x,w}\sum_{i\in I}\left(f_{i}x_{i}+r_{i}w_{i}\right)+E_{\Omega }\left[h\left(x,w,\xi \right)\right]$	Total cost	BB	Iteration	Node	EVNC	Stochastic	The model shows how renewables reduce load loss from production variability.
[72]	–	–	Reduce network	–	MCS	Node	EVNC EVFC	User behavior	Using conventional and fast charging, EVs can minimize the impact on the distribution network from charging.
[75]	MILP	$\mathrm{Min}\;\sum_{k=1}^{n}c_{k}x_{k}$	Total cost	BB	Iteration	Node	EVNC	Power demand	Solution quality varies with algorithm efficiency, problem size, and system requirements.
[76]	MCFP	$\mathrm{Min}\;z_{\left(q\right)}\sum_{p\in \beta }\sum_{k=1}^{t}q_{k}\psi_{p}\theta_{pk}$	Total cost	HA	CymDist	Network	EVFC	Trafic flow	M/M/s queuing system determines the optimal number of charging terminals.
*	MIP	$\mathrm{Min}\sum_{i=1}^{n}\sum_{j=1}^{n}w_{i}d_{ij}x_{ij}$	Total access cost	GA	DES (Arena)	Public (Node)	EVNC EVFC	Parking time/250–3750 EV	The variability in the type of charging modules leads to a reduction in waiting times, while the influx of electric vehicles (EVs) into the market dictates the strategic locations for new charging stations.

*This work, IP: Integer Programming, LP: Linear Programming, MIP: Mixed-Integer Programming, MILP: Mixed-Integer Linear Programming, MIQP: Mixed-Integer-Quadratic Programming, MINLP: Mixed-Integer Non-Linear Programming, CMC: Capacitated Maximal Coverage, MCLP: Maximum Coverage Location Problem, SP: Stochastic Programming, MCFP: Multicommodity Flow Problem, HA: Hungarian Algorithm, GA: Genetic Algorithm, PSO: Particle Swarm Optimization, MPDIPA: Modified Primal-Dual Interior Point Algorithm, WOA: Whale Optimization Algorithm, FWA: Floyd-Warshall Algorithm, LR: Lagrangian Relaxation DES: Discrete-Event Simulation, MCS: Monte Carlo Simulation, ABS: Agent-Based Simulation, EVFC: Electric Vehicle Fast Charger, EVNC: Electric Vehicle Normal Charger, PT: Parking Time, BES: Battery Exchange Stations, BB: Branch and Bound, SCP: Set Covering Problem, FCLP: Fixed-Charge Location Problem, O-D: Origin-Destination, OSSP: Optimal Siting and Sizing Problem, MP: Market Penetration.

In Ref. [[17], [18], [19], [20], [21], [22], [23], [24]], the p-median model emerges as a critical analytical tool for optimizing the spatial distribution of facilities, specifically aiming to minimize user travel distance. This model proves particularly efficacious in scenarios where the primary goal is to reduce the aggregate distance traversed for accessing services across a predetermined number of facilities within a designated area. Its utility is markedly pronounced in the development of cost-efficient EV charging infrastructures, where optimal siting is paramount to ensure accessibility across a broad spectrum of potential locations. The application of the p-median model to the siting of EVCS is extensively documented, with notable implementations highlighting its adaptability and relevance to current infrastructural challenges [19]. For instance, the investigation detailed in Ref. [20] introduces a novel methodology for identifying high-traffic areas as prime candidates for alternative energy charging stations, thereby facilitating a reduction in average refueling times. In a divergent approach, the study presented in Ref. [25]employs behavioral models to forecast EV charging demand, with an overarching aim of minimizing total travel distances. Moreover, ref. [23] delineates an optimization strategy to ascertain the optimal quantity, locations, and dimensions of EVCSs through the application of the Modified Primal-Dual Interior Point Algorithm. Concurrently, ref. [24] articulates a solution utilizing Mixed-Integer Nonlinear Programming and genetic algorithms to pinpoint the optimal locales and sizes for fast EVCSs. This strategy endeavors to mitigate development and electrification expenses alongside minimizing impacts on the electricity grid and reducing charging inefficiencies.

In Ref. [[26], [27], [28], [29], [30], [31], [32], [33], [34], [35], [36], [37]], the set covering location model and the maximal coverage location model are presented as pivotal methodologies in facility location optimization. The set covering model seeks to ascertain the minimal number of facilities required to comprehensively service an entire area without any limitations, whereas the maximal coverage model imposes constraints on the facility count to optimize service coverage. These models have been applied across a spectrum of facility types, evidencing their versatility and effectiveness. Ref. [30] integrates set covering with maximal coverage approaches, employing mixed-integer programming techniques to strategize the placement of diverse EVCS, including slow, fast, and exchange variants. This analysis elucidates that a heterogenous array of charging station types can yield more cost-effective solutions. Concurrently, ref. [32] advocates for an optimization model dedicated to minimizing the aggregate costs associated with EV charging infrastructures. Further exploration in Ref. [33] yields a solution to the maximal coverage dilemma by focusing on the strategic location of fast EVCSs within urban locales, navigating the challenges posed by fiscal limitations and traffic densities. Similarly, ref. [34] adopts a capacitated maximal coverage location problem model, striving to maximize total charging demand satisfaction within the constraints of budgetary and capacity limitations. Ref. [35] advances a methodology tailored for the optimization of charging station locations to accommodate mixed traffic flows. Additionally, ref. [23] innovates an optimization framework utilizing Geographic Information System (GIS) and Voronoi diagrams to efficiently service designated areas with the fewest stations feasible. Lastly, ref. [37] tackles the urban siting of fast charging stations through a linear programming relaxation-based algorithm, factoring in existing gasoline stations as potential sites for conversion or co-location. Collectively, these studies underscore the critical role of location optimization models in enhancing the accessibility and efficiency of EV charging infrastructure, thereby supporting the broader adoption of electric mobility solutions.

In Ref. [[38], [39], [40], [41], [42], [43], [44], [45], [46], [47], [48], [49], [50], [51], [52], [53]], the fixed-charge location problem is a model that considers the cost aspect of location problems, including land value and operating expenses. The fixed charge location model is used in various location problems such as large-scale facility location problems, distribution center problems, global scale problems, etc. Although this problem model is widespread, there are few studies related to the problem of EVCS locations. However, instead of writing a simple basic expression, the application of a modified constraint/objective function that takes into account the characteristics of the pricing infrastructure is often discussed. In Ref. [51] produced a swarm intelligence-based sample average approach by combining particle swarm optimization with the sample average approach, which they applied to the capacitated reliable facility location problem. Some studies have analyzed the location problems of plug-in electric vehicle (PHEV) charging stations. In Ref. [52] developed a mixed-integer programming model for PHEV charging stations based on specific geographic regions and parking lots. Ref. [53] addresses the deployment challenge of fast charging stations (FCS) for electric vehicles (EVs), considering elastic demand influenced by driving distance and waiting time. It proposes a fixed-point equation to model EV users' charging behavior, formulates the FCS deployment as a nonlinear integer problem, and employs a Genetic Algorithm-based heuristic for optimization. Simulation results demonstrate the efficacy of the proposed approach, highlighting the significance of aligning power grid capacity with charging demand for increased profit and reduced outage probability.

In Ref. [[54], [55], [56], [57], [58], [59], [60], [61], [62]], the Origin-Destination (O-D) trip-based model emerges as a sophisticated methodology that intricately considers both locational and routing dimensions of facility location challenges. This approach's utility is further augmented through the deployment of multiple O-D matrices, enabling a nuanced and realistic application of the model to a variety of scenarios. Specifically, reference [57] delves into evaluating the service capabilities and performance metrics of electric scooter charging stations employing a deterministic location allocation model paired with simulation techniques. Conversely, ref. [58] leverages integer programming to meticulously determine the strategic placement and requisite number of battery swapping stations, incorporating a comprehensive array of variables including battery range, multiplicity of routes, EV fleet size, and overarching operational constraints such as location and service capacity, alongside cost considerations. These investigations collectively underscore the model's robust applicability to resolving locational quandaries. Further, ref. [60] introduces a dual-tier programming framework aimed at pinpointing the optimal locales for EVCSs. This model distinguishes itself by optimally situating charging facilities to accommodate maximum vehicular flow at the macro level, while concurrently assimilating user-specific routing preferences and range constraints at the micro level. This duality facilitates a holistic optimization that not only addresses spatial efficiency but also user-centric requirements, thereby illustrating the comprehensive adaptability of the O-D trip-based model to the multifaceted domain of facility location optimization. Ref. [61], proposed an optimization method for electric vehicle charging station locations considering dynamic charging demand, using a Monte Carlo simulation for charging demand estimation and an improved whale optimization algorithm for better solution accuracy and convergence speed. Ref. [62], a mixed integer linear programming model has been formulated to maximize travel efficiency and preferences across the network under the constraint of a limited infrastructure investment budget. The effectiveness and efficiency of the proposed algorithms have been validated through numerical analyses on both synthetic and real-world networks.

In Ref. [[63], [64], [65], [66], [67], [68], [69], [70]], the dimensionality of EVCS emerges as a critical determinant of their operational efficiency. This necessitates a comprehensive analysis of the quantity, magnitude, and variants of EVCS to optimize functionality. Specifically, ref. [66] accentuates the focus on identifying the most advantageous positioning of EVCS and introduces an integrative framework aimed at addressing both locational and dimensional challenges. This model delineates the requisite number of EVCSs to curtail both construction and operational expenditures while incorporating vehicular traffic patterns as a limiting factor. Furthermore, ref. [70] elucidates the selection of prime locations designed to minimize the aggregate costs associated with the establishment of charging stations. This process leverages an M/M/x/s queue model to evaluate potential congestion at charging points and to ascertain the optimal count of charging apparatuses, selecting among a set of pre-evaluated sites for the installation of EVCS.

In Ref. [69,[71], [72], [73], [74], [75], [76]], the penetration and growth rates of EVs are identified as pivotal variables from an optimization standpoint. The projected quantity of EVs serves as a fundamental indicator for assessing EV charging demand. Consequently, the precision in forecasting EV quantities, particularly in the nascent phases, emerges as essential for the strategic deployment of EV charging infrastructure. The methodologies employed to gauge EV charging demand represent a focal area of scholarly inquiry. Notably, certain investigations have adopted predetermined metrics to estimate this demand. For instance, Ref. [71] applied a predefined proportion (10%) of the population and vehicle count to approximate the demand for battery swapping in Plug-in Hybrid EVs (PHEVs), further examining the spatial challenges associated with swapping station locations. Similarly, another study, as cited in Ref. [72], orchestrated an array of charger types within EV charging frameworks to ensure network stability, employing a constant EV penetration rate (20%) as determined through Monte Carlo simulations. Ref. [76] proposed a theoretical model that leverages OpenStreetMap data to efficiently develop fast charging station infrastructure. The model demonstrates its effectiveness in predicting EV demand and optimizing traffic flow through rerouting strategies. It offers scalable solutions for charging station providers in developing countries. Additionally, ref. [75] leveraged population size as a proxy for quantifying the demand at EVCSs, underscoring the significance of demographic considerations in infrastructure planning.

In this study, the p-median problem, addressed in Refs. [[17], [18], [19], [20], [21], [22], [23], [24]], was tackled for locating charging stations for electric vehicles. Similar to many studies in the literature, the primary aim is to minimize the total access cost. A genetic algorithm [77] was employed to solve the p-median problem. After determining the locations, the capacities of the charging stations were established by considering factors such as the market entry rates of electric vehicles [71,72], user charging behaviors [61,78], and queueing model [76]. A simulation model was then constructed using commercial software. This model serves as a significant tool for assessing the usage of electric vehicle charging stations and evaluating their performance.

**Fig. 2:**
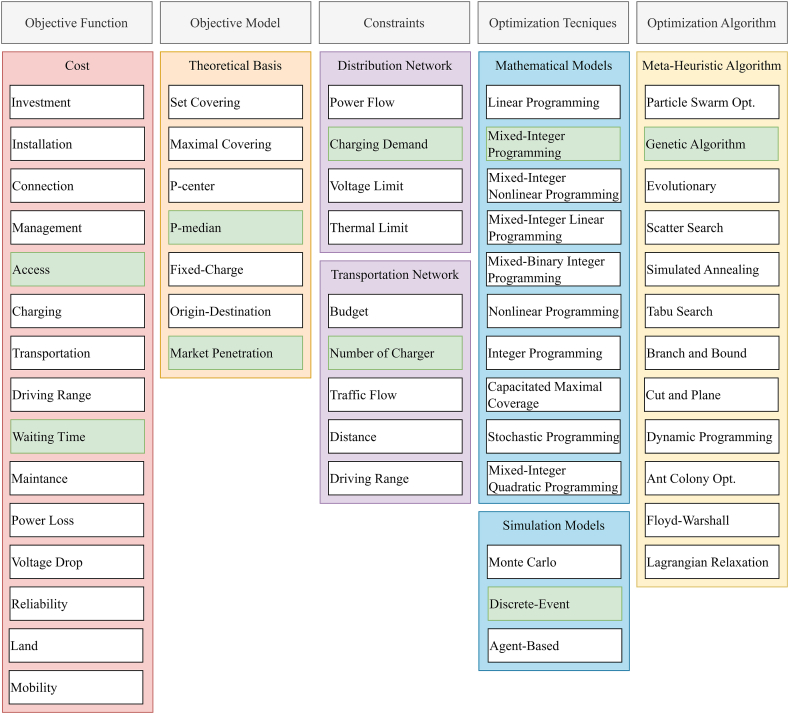
Methods for EVCS (green cell: this work)

Fig. 2 shows the categories in the literature and which methods are available. Considering the number of EVs entering the market, there is no other study that combines the p-median method, genetic algorithm and discrete-event simulation.

## 3 Methodology

### 3.1 P-median model

As discussed in Section 2, various models have been developed for the placement of charging facilities, with the p-median model being one of the most commonly used charging station location models. EVs are generally recharged while parked [79]. This implies that the demand for EV charging can be viewed as a series of points distributed across space. The p-median model is a typical node-based facility location model and is therefore particularly well-suited for processing such node-based charging demand. Consequently, we will utilize the p-median model to investigate how changes in the number of EVCS can affect station capacity and the type of charging module. In order to describe some of the inter-related elements in solving a p-median problem to optimality using Matlab software, we begin with the original model of ReVelle and Swain [18]. A form of their original formulation for the p-median problem involves the following notation:

(1) $$\mathrm{Min}\sum_{i\in I}\sum_{j\in J}w_{i}d_{ij}x_{ij}$$

s.t:

(2) $$x_{ij}\leq y_{j}\;i\epsilon I,j\epsilon J$$

(3) $$\sum_{j\in J}x_{ij}=1\;i\epsilon I$$

(4) $$\sum_{j\in J}y_{j}=p$$

(5) $$x_{ij}{,y}_{j}\;\epsilon \;\left\{0,1\right\}\;i\epsilon I,j\epsilon J$$

The p-median model aims to determine the locations of p facilities from among candidate locations by ensuring that each customer is served by one facility, thereby minimizing the transportation costs or weighted distances between customers and facilities. This situation can be mathematically represented by Eqs. (1)–(5). Eq. (1) states that the goal is to achieve the minimum total distance from the locations of all charging activities to the nearest charging stations; Eq. (2) specifies that only the charging station to be built can serve the nearby charging demand; Eq. (3) indicates that each demand point can only be met by one charging station; Eq. (4) the facility limitation, sets the exact number of facilities to be opened, which is predetermined by *p*. Eq. (5) ensures that the facility location and assignment decisions are constrained to binary choices, meaning they can only take on values of 0 or 1. This optimization framework is articulated through a set of mathematical formulations, where I and J represent the sets of charging demand points and candidate locations for charging stations, respectively. *w*_*i*_, the demand demand of the *i*-th demand point. Here, iϵI denotes a specific demand point, while jϵJ symbolizes a potential charging station site, typically associated with public parking lots. The distance between a demand point *i* and a candidate station *j* is denoted by *d*_*ij*_, which measures the spatial separation or the transportation cost from the demand point to the station. The decision-making process involves two binary decision variables: *y*_*j*_ and *x*_*ij*_. The variable *y*_*j*_ indicates whether location *j* has been selected for deploying a charging station (*y*_*j*_ = 1) or not (*y*_*j*_ = 0). Similarly, *x*_*ij*_ signifies whether the charging demand at point *i* is fulfilled by the station deployed at location *j* (*x*_*ij*_ = 1) or not (*x*_*ij*_ = 0). The objective is to deploy a target number *p* of charging stations in a manner that minimizes the total distance or transportation cost for all EV users to their nearest charging station, thereby enhancing accessibility and convenience. After determining the placement of charging stations using the p-median model, we ascertain the number of charging units for each station through a simulation approach. This encompasses both standard and fast charging units. Specifically, the number of charging units at a given station is largely determined based on charging demand.

### 3.2 Genetic algorithm

Genetic Algorithms (GAs), originally conceptualized by J.H. Holland in 1975, represent a class of evolutionary algorithms designed to solve optimization problems through mechanisms inspired by biological evolution, such as inheritance, mutation, selection, and crossover [77]. These algorithms have been extensively applied across a diverse range of disciplines, including engineering, economics, and management, demonstrating significant efficacy in addressing complex optimization challenges. Notably, GAs have proven particularly adept at navigating the intricacies of discrete facility location problems, a domain characterized by its NP-hard nature, indicating that no polynomial-time solution is known [80,81]. The foundational principle of GAs is to simulate the process of natural selection, wherein the most advantageous solutions-akin to the fittest individuals in a population-are iteratively selected for reproduction, thereby generating increasingly optimal solutions over successive generations. This methodology distinguishes itself from other optimization algorithms, such as hill climbing and simulated annealing algorithms, by offering a superior global search capability. This advantage stems from GAs' inherent design to explore a broader solution space, thereby enhancing the likelihood of identifying global optima for complex and large-scale optimization problems. Moreover, GAs are lauded for their conceptual simplicity, facilitating a more intuitive understanding of their operational mechanisms. This accessibility, combined with their robustness in solving diverse and complex problems, underscores the broad applicability of GAs in the optimization domain. A comprehensive elucidation of Genetic Algorithms and their application to optimization problems can be found in the seminal works by Holland [77] and subsequent studies [80,81].

**Fig. 3:**
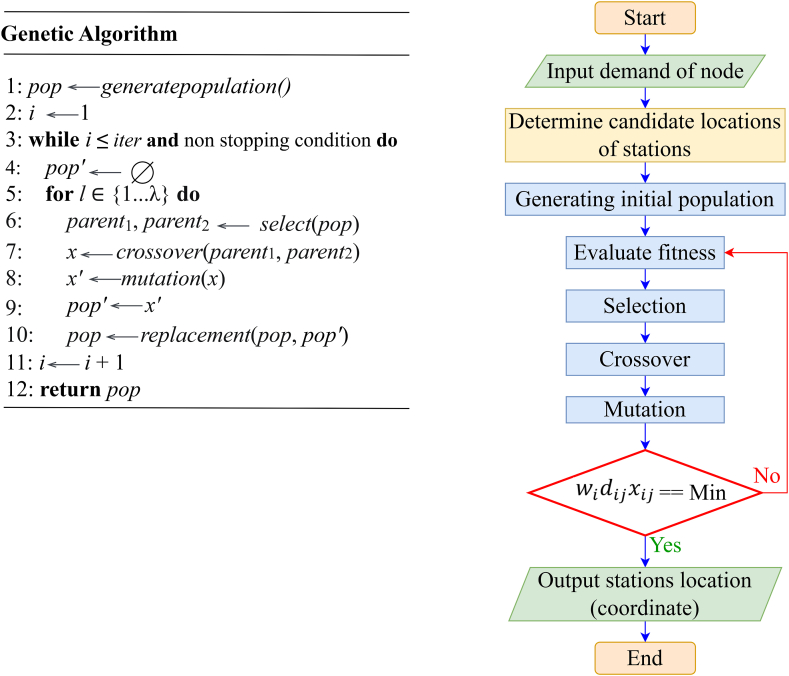
Pseudocode and flow chart of solving genetic algorithm.

In the study, Genetic Algorithm was coded in Matlab to find global optimal solutions. In Matlab, the GA function needed to set some parameters such as fitness function, number of elements of the variable, constraint, variable range, and variable type. A pseudocode and flowchart summarizing our GA selection algorithm is shown in Fig. 3.

The initial phase randomly selects p-medians for the first individual and sets initial fitness values. Fitness evaluation is then performed based on an objective function, considering node-to-median assignments. A node-median assignment method proposed by Correa et al. [82] is adapted. The process iteratively improves solutions by recalculating median points and reassigning nodes if fitness improves. The best solution among initial solutions becomes the population's best. The process repeats for the population size. The selection operator is then applied to determine individuals for the next generation, increasing iteration count by one.

## 4 Results and discussion

The predominant barriers impeding the universal adoption of EVs encompass the prohibitive costs associated with EVs, their constrained operational range, and the insufficiently developed charging infrastructure. With advancements in EV battery technologies and the subsequent expansion of the EV market, the establishment of charging stations tailored to meet the energy demands of these vehicles becomes imperative. The strategic siting of such charging facilities necessitates a comprehensive alignment with anticipated charging demand and vehicular traffic patterns. Furthermore, the design and distribution of charging stations must be informed by an in-depth analysis of the local electrical distribution network to ensure operational efficiency and sustainability. In the strategic planning process for charging infrastructure, it is essential to integrate these facilities within the broader urban development and road network strategies to foster a coherent and functional transportation ecosystem. The service range of each charging station should be determined based on rigorous criteria to effectively serve the needs of the EV user population. Additionally, foresight into the future trajectories of EV adoption and technological innovation is crucial in the planning phase, ensuring that the charging infrastructure remains resilient and adaptive to evolving transportation paradigms.

**Fig. 4:**
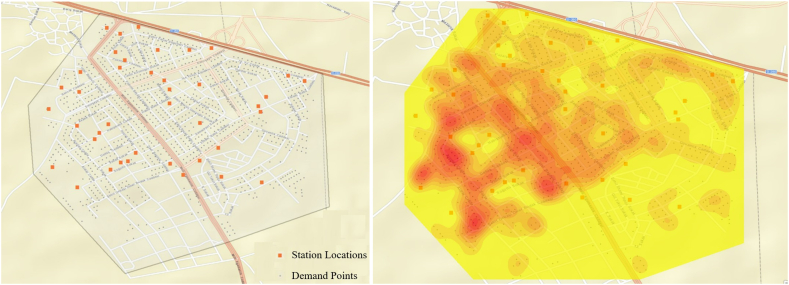
Demand points, potential station points and demand density map.

This study consists of two main parts. In the first part, the problem of selecting charging station locations is addressed using a genetic algorithm. In the second part, the capacity planning for charging stations is determined through a simulation study. Prior to commencing the study, both the demand points and potential station locations were identified. These demand and station points are treated as coordinates to be utilized in the algorithms. In this study, a total of 1035 demand points and 40 station locations were identified. These points are depicted in Fig. 4., facilitated by a geographic information system. Given that the sequence of station openings is a significant criterion, a demand density map was generated to visualize areas with dense demand points, as shown in Fig. 4. The density map illustrates where the initial stations should be established once EVs become available in the automotive market.

### 4.1 Station placement with genetic algorithm

Genetic algorithms are utilized to tackle optimization challenges, initiating with a process that subjects a population to a selection phase using an objective function. Following this phase, individuals within the population are paired to undergo crossover. Once the crossover is completed, a mutation process is introduced, potentially altering the individuals. This cycle persists until an adequate solution is discovered. In this study, the structure of GA is concisely outlined as follows: Initially, an initial population is formed by equally distributing points from a total of *i* demand points to *j* facilities. The population is then evaluated through a fitness function for selection. Subsequently, a neighborhood structure, comprising several replacement operations determined by specific probabilities, is applied. These operations include substituting a plant location with any demand point, exchanging facility locations among themselves, and swapping demand points. Afterward, the individuals undergo the crossover process, followed by the application of mutation to the population. If the iteration count is below the desired threshold, the process reverts to the second step; otherwise, it progresses. The optimal chromosome identified is deemed the best outcome.

**Fig. 5:**
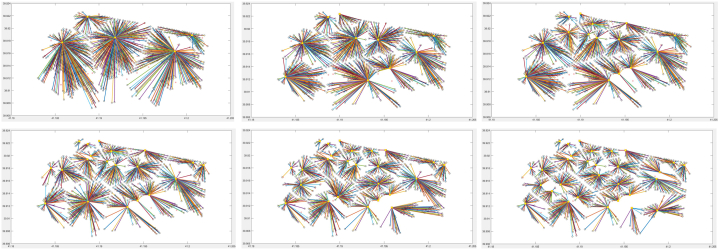
Roulette wheel, tournament selection and random solution method with 5, 10, 15, 20, 25, and 30 station locations.

For the solution of the problem, the population size was taken as 100, the crossover rate as 0.80, the mutation rate as 0.05 and the number of iterations as 100. When the problem was solved according to these parameters, results as shown in Table 2 were found. The genetic algorithm was run in Matlab®2020b software and on a 12 GB RAM PC Intel® CoreTM i7 CPU 2.00 GHz processor. Roulette wheel, tournament selection, random solution method and 30 station locations are shown in Fig. 5 and the results of these methods are given in Fig. 8. The yellow dots in the figure represent the 30 charging service stations and the small circle dots represent the charging demands. The total cost was found to be 8.321.464,90 units for all three methods. The cost graph is as shown in Fig. 7. When the results of the program are examined, it is found that there is no difference.

**Table 2:** Genetic algorithm results for station placement without capacity.

Method	Number of Stations	Iteration	Number of Functions	Best Cost (unit)	Percentage Increase (%)	Time (sec)
	5	100	11100	480.387,60		31,569
	10	100	11000	1.370.818,21	185,4	30,614
Roulette Wheel Selection	15	100	11000	2.363.767,72	72,4	34,884
	20	100	11000	3.555.306,51	50,4	30,729
	25	100	11000	5.541.205,52	55,9	35,569
	30	100	11000	8.321.464,90	50,2	30,700
	5	100	11100	480.387,60		32,273
	10	100	11000	1.370.818,21	185,4	33,282
Tournament Selection	15	100	11000	2.363.767,72	72,4	31,105
	20	100	11000	3.555.306,51	50,4	31,286
	25	100	11000	5.541.205,52	55,9	32,029
	30	100	11000	8.321.464,90	50,2	30,700
	5	100	11100	480.387,60		32,023
	10	100	11000	1.370.818,21	185,4	30,458
Random Selection	15	100	11000	2.363.767,72	72,4	32,572
	20	100	11000	3.555.306,51	50,4	30,449
	25	100	11000	5.541.205,52	55,9	30,968
	30	100	11000	8.321.464,90	50,2	31,143

**Fig. 6:**
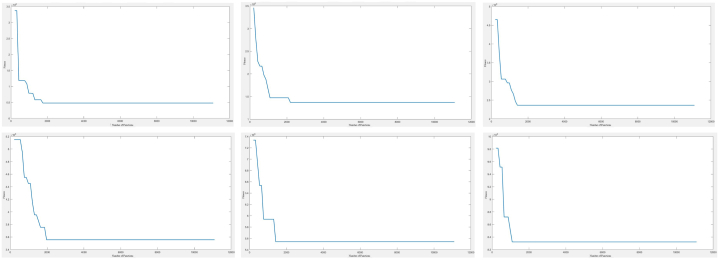
Convergence curve for 5, 10, 15, 20, 25, and 30 station locations.

**Fig. 7:**
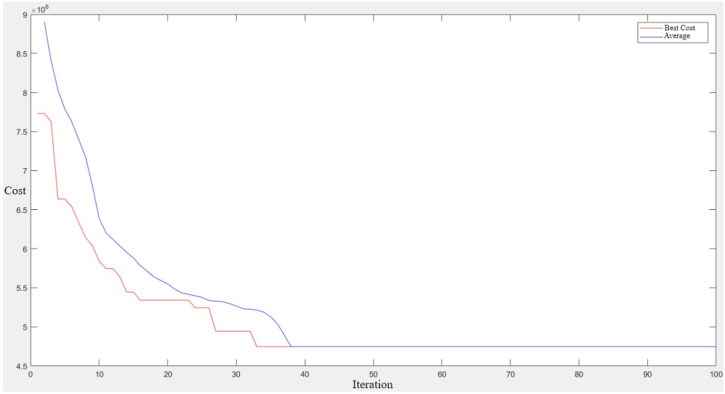
Computational burden.

GA was used to solve the site selection model and the costs were calculated. From Table 2 and Fig. 6., we can see the change in the costs of the objective function value for 5, 10, 15, 20, 25 and 30 stations when different numbers of charging stations are installed. After 15 stations, the increase in costs remained at 50%. This result implies that the increase in the number of stations will keep the costs constant in percentage after a while and is supported by the results found in Ref. [83]. Also, Fig. 7 shows the convergence curve where the blue line represents the average cost convergence process and the red line represents the best cost convergence process. As can be seen from Fig. 7., the best cost is solved in 33 iterations.

**Fig. 8:**
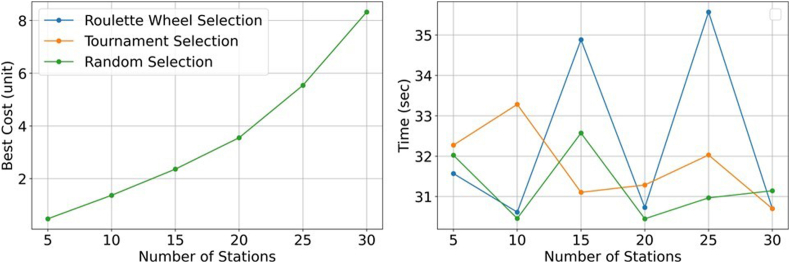
Cost graph for 30 station locations with roulette wheel, tournament and random selection method.

When the results of the program are examined, it is found that there is no difference between the three methods solved in terms of the locations and costs of the stations.

**Fig. 9:**
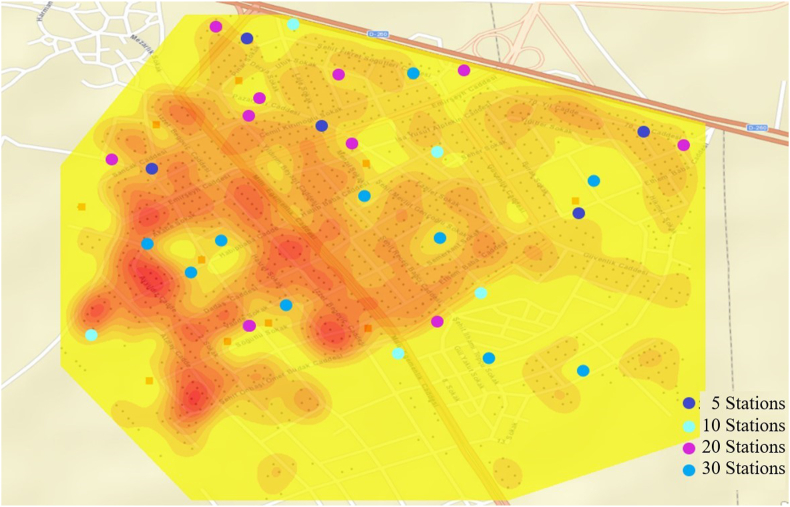
Opening sequence of charging station.

Since the acceptance and use of EVs by car users will take place within a certain period of time, the opening of charging stations will be parallel to this period. Considering this process in solving the problems, the number and location of the first stations to be opened is also an important criterion. As a result of the results given above, it was determined where the number of stations between 5 and 30 stations should be opened and it is shown in Fig. 9.

In section 4.2 of the study, a simulation model was developed to determine where to locate EVCSs based on demand and how many and which type of EV charging units should be located at the station, taking into account the current location. As part of this, it is aimed to reduce EV traffic density and waiting times of the facilities.

### 4.2 Simulation model

Arena simulation software serves as a sophisticated tool designed to forecast, scrutinize, and authenticate system or process strategies for their enhanced and optimized operational performance. It employs discrete event modeling to facilitate the optimization of intricate processes with relative ease. The software adopts a flowchart modeling approach, wherein various process variables are delineated based on a range of predefined or user-specified functions or statistical distributions. Capable of articulating the dynamics of complex systems, including those characterized by finite resources and intricate interactions, it can accurately model virtually any variable process. Its capability to present the simulation study of the entire process in either two-dimensional or three-dimensional visuals significantly aids in the elucidation of the simulation outcomes. Furthermore, the software is adept at handling multiple variables over time, enabling the conduct of various statistical analyses and the generation of comprehensive reports detailing all findings.

**Fig. 10:**
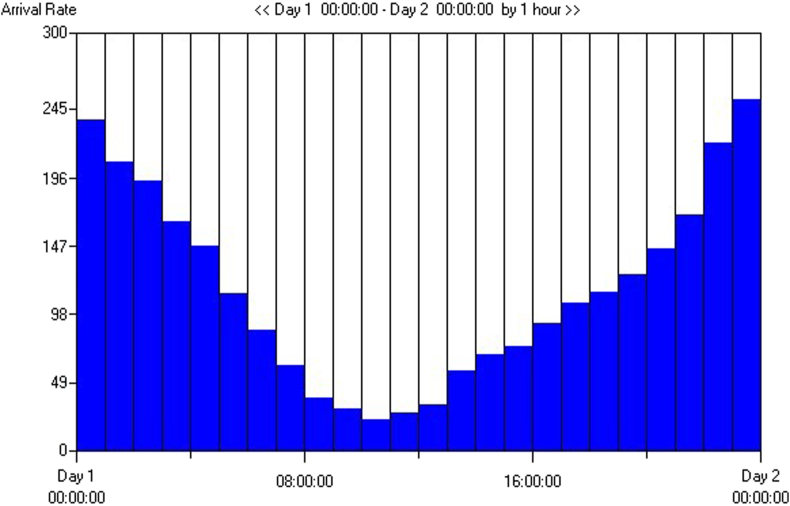
Time between arrivals of EVs users at stations.

The system in question is designed to evaluate the necessary number and types of charging stations required to meet the charging demands of EVs. It incorporates various inputs, including the EVs themselves and the charging stations. The primary objective of this system is to ascertain the optimal number of EV charging stations and the specific types of charging modules required to efficiently service EVs. The system comprises several key elements, such as the stations, the vehicles, and the charging modules, all of which interact to influence outcomes like vehicle waiting times. These times are affected by the capacities of the charging stations and the charging module types utilized. The system is parameterized by the number of EVs, charging stations, and charging modules. Its performance is evaluated based on criteria such as station occupancy rates, the number of vehicles awaiting service, and the waiting times of these vehicles. The output of the system is the number of EVs that have their charging needs satisfactorily met. Underpinning the simulation model are assumptions about the variability in the arrival times of EVs throughout the day, segmented into 24-h periods, with vehicle arrivals fluctuating according to the different times of day to reflect the varied schedules of EV users. This approach allows for a tailored simulation that accommodates the dynamic nature of EV charging demand, as depicted in the model. The different inter-arrival times were created in accordance with the working hours of the EV users and shown in Fig. 10. According to Fig. 10., it is observed that the highest demand for charging by electric vehicle users occurs between 16:00 and 24:00. These user behaviors support that the peak hours are after working hours, as ref [61,78] did in their study.

The simulation study is premised on the scenario where EVs prioritize locating an available charging station, and in instances where all stations are occupied, the EV will depart without charging. Given the insufficient number of EVs in the region to gather comprehensive data, it is assumed their arrivals at the stations are random. This study is intentionally designed to allow for future updates, which could include considerations of the distances between stations and user data. The initial phase of this simulation focuses on the creation of EVs as arriving entities, involving the generation of assets and the assignment of time variables to each for subsequent processing and integration into the simulation modules. The station selection algorithm commences with each vehicle determining its destination, followed by the selection of the most suitable station according to a distributed station selection rule. Upon arrival, if an idle charging unit is available, the vehicle immediately starts charging; otherwise, it queues up and awaits its turn based on a First-In, First-Out (FIFO) queue management system. Once charging is complete, the vehicle departs from the station. Additionally, the status of each station is periodically reviewed in alignment with vehicle routing controls, to efficiently manage and optimize station usage. This approach sets a foundation for a detailed analysis of the dynamics of EVCS utilization, with provisions for integrating more complex factors in future research developments.

This framework lays the groundwork for a comprehensive analysis of EVCS dynamics, with the flexibility to incorporate more complex variables in future research iterations. The simulation model was run for 5,10,15 and 20 stations for 24 h and 30 repetitions. While creating the scenarios, the number of vehicles arriving at the station and the number of charging units were changed to find the appropriate number of charging units. The aim of the simulation is to minimize the number of vehicles that cannot be charged and leave the station. Table 3 and Table 4 shows the scenario created for the stations.

**Table 3:** Number of normal and fast charging units in the scenarios created for 5 stations.

Scenario Properties		Control										Response	
Name	Number of Vehicles	Station 1		Station 2		Station 3		Station 4		Station 5		Not Charged	Demand Fulfillment Rates (%)
		Normal	Fast	Normal	Fast	Normal	Fast	Normal	Fast	Normal	Fast		
Scenario 1	1000	20	15	20	15	20	15	20	15	20	15	10	99,00
Scenario 2	1250	20	15	20	15	20	15	20	15	20	15	15	98,80
Scenario 3	1500	25	25	20	15	25	15	25	25	25	25	12	99,20
Scenario 4	2000	25	15	20	15	20	15	20	15	20	15	55	97,25
Scenario 5	2250	25	15	20	15	25	20	25	15	25	20	47	97,91
Scenario 6	2500	20	25	20	30	20	20	20	20	20	15	7	99,72
Scenario 7	2750	20	20	20	15	20	15	20	25	20	10	237	91,38
Scenario 8	3000	20	25	20	25	20	25	20	15	20	15	11	99,63
Scenario 9	3250	25	15	25	15	25	15	25	10	25	5	466	85,66
Scenario10	3500	20	30	20	30	20	30	20	20	20	20	2	99,94

**Table 4:** Number of vehicles that cannot be charged for 10 stations.

Scenario Properties			Control	Response	
Name	Number of Stations	Reps	Number of Vehicles	Not Charged	Demand Fulfillment Rates (%)
Scenario 1	10	30	250	0	100
Scenario 2	10	30	500	0	100
Scenario 3	10	30	750	0	100
Scenario 4	10	30	1000	0	100
Scenario 5	10	30	1250	0	100
Scenario 6	10	30	1500	0	100
Scenario 7	10	30	1750	0	100
Scenario 8	10	30	2000	0	100
Scenario 9	10	30	2250	0	100
Scenario 10	10	30	2500	31	98,76
Scenario 11	10	30	2750	93	96,62
Scenario 12	10	30	3000	159	94,70
Scenario 13	10	30	3250	227	93,02
Scenario 14	10	30	3500	258	92,63
Scenario 15	10	30	3750	258	93,12

For 5 stations, the longest queue waiting time was 1.2 min at the second station, which was opened. For 10 stations, the longest queue waiting time was 0.5 min at the sixth station. For 15 and 20 stations, it was found that there were no vehicles that could not be charged. When the information on the hours of the day between which the charged vehicles were charged was analyzed, it was found that there was a density between 21:00–07:00 for all stations. In the first hours of the day and until the end of working hours, no queue was observed in the system. In line with the simulation results above, scenarios that minimize the number of vehicles that cannot be charged by changing the number of vehicles and the number of charging units were tried to be found in the model. While preparing the scenarios, the total vehicle capacities of the station were taken into consideration. Table 3 below shows the number of stations and the coordinates of the stations to be opened, the number of charging units to be opened in the first installation and their alternatives for 3.405 vehicles and 1.035 demand points. In addition to the first installed units, the alternatives that the system can operate with two different scenarios are indicated.

The results show that an average of 3.257 vehicles entered the system for 5 stations and 1.921 of these vehicles were charged in fast charging units, 118 vehicles in normal charging units and 1.217 vehicles left the system without being charged. The peak hours of the day are between 21:00 and 07:00. Alternative scenarios were created to minimize the number of non-rechargeable vehicles. When the scenarios are examined, the first 5 station locations were determined after the first EVs were started to be used and the number of units that should be opened at these stations was determined. Table 5 shows the scenario created for the stations. For example, for the station to be opened at coordinates (39,922258-41,187756), the number of normal charging units to be installed first is 45 and the number of fast charging units is 40. In alternative scenarios, this number is 25 for normal charging units and 55 for fast charging units. In another alternative scenario, it is 20 for normal level charging units and 60 for fast charging units. This situation reveals that the scenarios can be further multiplied. As the number of stations increases, it is seen that the number of charging units required at the charging stations decreases. For 5 stations (39,915500-41,194365) coordinates, the number of normal charging units is 60 and the number of fast charging units is 70 in the first installation, while the number of normal units in this station is 2 and the number of fast units is 13 when 20 stations are opened. In the simulated study for various number of stations, the total number of vehicles arriving at the system, queue length, waiting times and charging times provide important information about the times of the day when congestion occurs.

**Table 5:** Number of stations and charging units.

Number of Stations	Coordinates of Stations		Initial Setup		Alternative Unit Numbers			
	X Coordinate	Y Coordinate	Normal Unit	Fast Unit	Normal Unit	Fast Unit	Normal Unit	Fast Unit
5	39,922258	41,187756	45	40	25	55	20	60
	39,919953	41,189032	55	60	60	40	50	50
	39,922325	41,189987	45	55	35	45	60	25
	39,915500	41,194365	60	70	40	80	75	60
	39,919080	41,190892	85	75	95	25	85	65
10	39,922258	41,187756	25	25	20	28	22	35
	39,919953	41,189032	30	30	25	25	25	30
	39,918244	41,194329	27	25	35	32	33	38
	39,916700	41,198514	30	22	40	12	30	12
	39,917352	41,199052	19	18	20	25	21	23
	39,918476	41,201809	22	32	28	27	28	29
	39,916296	41,198670	24	32	21	18	30	25
	39,912201	41,188007	25	26	19	30	30	23
	39,919130	41,185866	33	34	25	35	36	36
	39,918015	41,184535	36	45	26	40	40	43
15	39,916503	41,183614	23	24	20	21	22	24
	39,918244	41,194329	25	25	35	15	30	20
	39,916700	41,198514	30	30	35	35	20	30
	39,917352	41,199052	19	21	18	18	16	19
	39,910938	41,185659	16	16	13	18	12	18
	39,915417	41,187851	17	18	16	14	18	18
	39,913385	41,189791	15	15	10	25	25	25
	39,912619	41,192256	26	28	19	21	23	28
	39,911825	41,193179	24	24	25	25	20	30
	39,912826	41,194336	23	25	20	20	24	26
	39,913744	41,195666	22	27	20	40	36	10
	39,911286	41,198756	36	21	20	45	25	20
	39,912201	41,188007	12	18	11	25	10	25
	39,912701	41,188737	24	24	28	28	26	24
	39,912784	41,189252	18	18	16	19	17	20
20	39,919080	41,190892	15	12	10	20	11	20
	39,920829	41,195184	11	10	13	15	8	17
	39,918868	41,200634	11	8	9	8	7	13
	39,919424	41,188671	10	5	10	6	12	5
	39,918244	41,194329	13	9	8	15	7	16
	39,916700	41,198514	8	3	7	5	7	7
	39,917352	41,199052	6	2	4	3	4	5
	39,919953	41,189032	4	10	5	12	3	15
	39,922325	41,189987	12	5	11	6	10	7
	39,918519	41,191836	13	5	12	6	12	6
	39,919080	41,190892	10	16	9	15	8	17
	39,913385	41,189791	7	4	6	2	8	3
	39,912619	41,192256	8	3	10	1	5	5
	39,911825	41,193179	9	5	8	8	6	8
	39,916700	41,198514	10	12	9	12	10	10
	39,917352	41,199052	2	8	1	10	2	10
	39,918476	41,201809	10	8	10	10	12	12
	39,912201	41,188007	7	11	7	10	6	9
	39,915500	41,194365	2	13	5	4	9	8
	39,919130	41,185866	10	4	15	0	1	10

## 5 Conclusions

EVs play a significant role in the future of the automotive industry as a green and innovative mode of transportation. For these vehicles to be widely used, supporting infrastructures such as EVCSs are required. As highlighted in Ref. [25], strategically positioning public charging stations presents a cost-efficient strategy for incorporating EVs into the transportation market. The design, functionality, and placement of these charging stations are pivotal factors that will directly influence EV utilization and the expansion of this sector. The main achievements focused on in this article are stated below:

Firstly, a suitable location model for EVCS has been created based on demand. While creating the location model, only public locations were considered. It is stated in the model's constraint conditions that the number of charging stations to be opened is limited to a predetermined number *p*. This limits the maximum number of facilities that can be selected, making the problem more manageable.

Secondly, by entering the demand and potential charging station coordinates into the model, the location model becomes practical and effective. An example has been used to prove that the location model is practical and effective. The optimal location of charging stations, as in the study [25,41] has yielded positive outcomes, including average access distance and total access cost.

Lastly, we make reasonable suggestions regarding the location of EVCS. This article primarily selects the locations, capacities, and types of charging units at the station. In this process, the article analyzes the impact of different numbers of EVCS on consumer demand, concluding that balancing the waiting time at EVCS by changing the type of unit at the stations reduces it, as in the studies of [34]. Additionally, Ref. [72,74] optimizing the normal and fast charging modules of EVs demonstrates that it can effectively reduce the burden on the distribution network caused by EVs.

The study plans the location of the EVCS and the type of charging unit inside the station, considering the gradual entry of EVs into the market and consumers' charging behavior. Due to the lack of an EVCS system in Turkey, there are some limitations in the variables and factors in this problem. Generally, this article mainly includes the following advanced studies:

In the conceptualization of the EVCS location problem, it is imperative to integrate both the demand dynamics and the quantitative expansion of charging stations to orchestrate an optimal locational strategy. This strategy should account for installation expenses as well as the existing electric grid infrastructure. The completion of a comprehensive charging network, maximizing the utility of extant charging facilities, ensuring ease of access to services, and delivering societal benefits, should guide the trajectory of future research endeavors. Additionally, the application of heuristic algorithms, including Particle Swarm Optimization and Tabu Search, represents a significant area for future investigation, providing robust solutions to the location optimization problem.

## Funding statement

This research did not receive any specific grant from funding agencies in the public, commercial, or not-for-profit sectors.

## Data availability statement

Data will be made available on request.

## Declaration of interest's statement

The authors declare that they have no known competing financial interests or personal relationships that could have appeared to influence the work reported in this paper.

## CRediT authorship contribution statement

**Serdar Çelik:** Writing – review & editing, Visualization, Software, Resources, Project administration, Methodology. **Şeyda Ok:** Visualization, Software, Resources.

## Declaration of competing interest

The authors declare that they have no known competing financial interests or personal relationships that could have appeared to influence the work reported in this paper.
