# Supplementary material for: Electric vehicle charging stations: Model, algorithm, simulation, location, and capacity planning
Source: Heliyon. 2024 Apr 6;10(7):e29153. doi: 10.1016/j.heliyon.2024.e29153 (PMC11016620; doi:10.1016/j.heliyon.2024.e29153)
Supplement: Multimedia component 2 [file mmc2.docx]

## Demand Points Coordinate and Demand

| **X Coordinate** | **Y Coordinate** | **Demand** | **X Coordinate** | **Y Coordinate** | **Demand** |
| --- | --- | --- | --- | --- | --- |
| 39,922605 | 41,188120 | 2 | 39,920699 | 41,189480 | 2 |
| 39,922490 | 41,187669 | 3 | 39,921933 | 41,190382 | 1 |
| 39,922144 | 41,187412 | 2 | 39,921884 | 41,190682 | 2 |
| 39,922013 | 41,187755 | 3 | 39,921785 | 41,190940 | 1 |
| 39,921914 | 41,187219 | 2 | 39,921719 | 41,191197 | 2 |
| 39,921733 | 41,187541 | 2 | 39,921588 | 41,191519 | 1 |
| 39,921733 | 41,187541 | 3 | 39,921505 | 41,191776 | 2 |
| 39,921503 | 41,187326 | 2 | 39,921407 | 41,192141 | 1 |
| 39,921963 | 41,188914 | 1 | 39,921357 | 41,192399 | 1 |
| 39,921749 | 41,188378 | 2 | 39,921259 | 41,192763 | 2 |
| 39,921585 | 41,188184 | 3 | 39,921193 | 41,192999 | 2 |
| 39,921363 | 41,188077 | 3 | 39,921110 | 41,193278 | 2 |
| 39,921190 | 41,187927 | 3 | 39,921078 | 41,193514 | 2 |
| 39,920976 | 41,187745 | 3 | 39,921012 | 41,193858 | 1 |
| 39,920811 | 41,187573 | 3 | 39,920929 | 41,194115 | 1 |
| 39,920573 | 41,187433 | 3 | 39,920847 | 41,194394 | 1 |
| 39,921552 | 41,188742 | 2 | 39,921703 | 41,190124 | 1 |
| 39,921363 | 41,188592 | 2 | 39,921604 | 41,190382 | 2 |
| 39,921182 | 41,188410 | 2 | 39,921522 | 41,190704 | 1 |
| 39,921017 | 41,188249 | 2 | 39,921456 | 41,190982 | 1 |
| 39,920844 | 41,188109 | 2 | 39,921357 | 41,191390 | 2 |
| 39,920663 | 41,187981 | 2 | 39,921275 | 41,191691 | 3 |
| 39,920441 | 41,187798 | 2 | 39,921160 | 41,191991 | 2 |
| 39,922128 | 41,190030 | 1 | 39,921061 | 41,192291 | 1 |
| 39,921610 | 41,189483 | 1 | 39,920847 | 41,192163 | 2 |
| 39,921297 | 41,189054 | 1 | 39,920929 | 41,191841 | 3 |
| 39,921223 | 41,189568 | 2 | 39,921012 | 41,191562 | 2 |
| 39,920614 | 41,188775 | 3 | 39,921061 | 41,191261 | 1 |
| 39,920913 | 41,189910 | 2 | 39,921176 | 41,190875 | 2 |
| 39,920699 | 41,189995 | 2 | 39,921259 | 41,190575 | 1 |
| 39,920502 | 41,190103 | 3 | 39,921357 | 41,190317 | 2 |
| 39,920304 | 41,190189 | 3 | 39,920946 | 41,190403 | 2 |
| 39,920090 | 41,190274 | 2 | 39,920699 | 41,190446 | 1 |
| 39,919893 | 41,190360 | 2 | 39,920502 | 41,190553 | 1 |
| 39,919679 | 41,190446 | 1 | 39,920288 | 41,190639 | 2 |
| 39,919777 | 41,190017 | 2 | 39,920074 | 41,190725 | 1 |
| 39,919975 | 41,189888 | 1 | 39,919794 | 41,190832 | 2 |
| 39,920139 | 41,189781 | 2 | 39,919563 | 41,190897 | 3 |
| 39,920337 | 41,189695 | 3 | 39,920814 | 41,190832 | 2 |
| 39,920502 | 41,189566 | 2 | 39,920600 | 41,190918 | 2 |

## Demand Points Coordinate and Demand (continued)

| **X Coordinate** | **Y Coordinate** | **Demand** | **X Coordinate** | **Y Coordinate** | **Demand** |
| --- | --- | --- | --- | --- | --- |
| 39,920370 | 41,191025 | 3 | 39,920081 | 41,187113 | 1 |
| 39,920123 | 41,191133 | 2 | 39,920134 | 41,187435 | 2 |
| 39,919909 | 41,191197 | 1 | 39,919887 | 41,187612 | 4 |
| 39,919728 | 41,191218 | 3 | 39,919834 | 41,187307 | 5 |
| 39,920139 | 41,191605 | 2 | 39,919525 | 41,188052 | 4 |
| 39,920255 | 41,191862 | 2 | 39,919336 | 41,188213 | 3 |
| 39,920353 | 41,192141 | 1 | 39,919163 | 41,187886 | 2 |
| 39,919876 | 41,191648 | 3 | 39,919324 | 41,187725 | 2 |
| 39,919975 | 41,191970 | 2 | 39,919492 | 41,187602 | 4 |
| 39,920041 | 41,192227 | 2 | 39,919644 | 41,187457 | 3 |
| 39,920995 | 41,192742 | 3 | 39,919017 | 41,188460 | 4 |
| 39,920814 | 41,192978 | 3 | 39,918820 | 41,188626 | 4 |
| 39,920683 | 41,192635 | 3 | 39,918627 | 41,188755 | 4 |
| 39,920584 | 41,193128 | 3 | 39,918458 | 41,188943 | 3 |
| 39,920370 | 41,193193 | 2 | 39,918285 | 41,189066 | 4 |
| 39,920460 | 41,192666 | 2 | 39,918129 | 41,188728 | 3 |
| 39,920221 | 41,192752 | 1 | 39,918339 | 41,188567 | 4 |
| 39,920857 | 41,195226 | 1 | 39,918548 | 41,188358 | 7 |
| 39,920413 | 41,194292 | 1 | 39,918812 | 41,188187 | 7 |
| 39,920310 | 41,194013 | 1 | 39,919177 | 41,188889 | 7 |
| 39,920215 | 41,193740 | 1 | 39,919267 | 41,189146 | 3 |
| 39,920117 | 41,193439 | 2 | 39,919358 | 41,189415 | 2 |
| 39,920047 | 41,193171 | 2 | 39,919432 | 41,189672 | 2 |
| 39,919919 | 41,192882 | 1 | 39,918975 | 41,189087 | 2 |
| 39,919602 | 41,193085 | 1 | 39,919066 | 41,189350 | 2 |
| 39,919717 | 41,193332 | 2 | 39,919140 | 41,189575 | 2 |
| 39,919792 | 41,193600 | 1 | 39,919218 | 41,189854 | 3 |
| 39,919878 | 41,193842 | 2 | 39,918654 | 41,189362 | 2 |
| 39,919985 | 41,194115 | 2 | 39,918732 | 41,189619 | 4 |
| 39,920080 | 41,194405 | 1 | 39,918835 | 41,189893 | 2 |
| 39,919434 | 41,192588 | 2 | 39,918918 | 41,190139 | 3 |
| 39,919343 | 41,192347 | 1 | 39,918996 | 41,190391 | 2 |
| 39,919253 | 41,192084 | 1 | 39,919066 | 41,190574 | 3 |
| 39,919170 | 41,191837 | 2 | 39,918416 | 41,189624 | 3 |
| 39,919117 | 41,191558 | 3 | 39,918502 | 41,189887 | 3 |
| 39,919380 | 41,191381 | 2 | 39,918580 | 41,190129 | 2 |
| 39,919475 | 41,191644 | 2 | 39,918691 | 41,190386 | 4 |
| 39,919569 | 41,191886 | 2 | 39,918765 | 41,190633 | 4 |
| 39,919668 | 41,192159 | 2 | 39,918876 | 41,190906 | 5 |
| 39,919746 | 41,192401 | 1 | 39,919935 | 41,185940 | 8 |

## Demand Points Coordinate and Demand (continued)

| **X Coordinate** | **Y Coordinate** | **Demand** | **X Coordinate** | **Y Coordinate** | **Demand** |
| --- | --- | --- | --- | --- | --- |
| 39,919849 | 41,186284 | 8 | 39,918010 | 41,186235 | 6 |
| 39,919684 | 41,186648 | 7 | 39,917759 | 41,186449 | 6 |
| 39,919441 | 41,186809 | 6 | 39,917471 | 41,186691 | 4 |
| 39,919441 | 41,186809 | 8 | 39,917249 | 41,186900 | 5 |
| 39,918984 | 41,187199 | 4 | 39,917130 | 41,186659 | 6 |
| 39,918823 | 41,186899 | 8 | 39,917348 | 41,186460 | 2 |
| 39,919017 | 41,186673 | 6 | 39,917661 | 41,186213 | 3 |
| 39,919235 | 41,186496 | 4 | 39,917895 | 41,185993 | 5 |
| 39,919453 | 41,186330 | 4 | 39,917249 | 41,186224 | 4 |
| 39,919618 | 41,185997 | 4 | 39,917019 | 41,186449 | 4 |
| 39,919692 | 41,185751 | 3 | 39,917643 | 41,185400 | 4 |
| 39,919177 | 41,185411 | 3 | 39,917367 | 41,185614 | 5 |
| 39,918902 | 41,185459 | 3 | 39,917092 | 41,185834 | 4 |
| 39,918663 | 41,185507 | 4 | 39,916828 | 41,186054 | 3 |
| 39,918466 | 41,185534 | 3 | 39,916709 | 41,185818 | 4 |
| 39,918256 | 41,185550 | 4 | 39,916964 | 41,185646 | 5 |
| 39,918256 | 41,185550 | 3 | 39,917388 | 41,184933 | 5 |
| 39,918289 | 41,185212 | 7 | 39,916857 | 41,185394 | 5 |
| 39,918289 | 41,185212 | 4 | 39,916585 | 41,185587 | 5 |
| 39,918972 | 41,184810 | 5 | 39,916458 | 41,185378 | 5 |
| 39,918696 | 41,184724 | 4 | 39,916355 | 41,185148 | 6 |
| 39,918659 | 41,184982 | 5 | 39,916622 | 41,185008 | 6 |
| 39,918601 | 41,184241 | 4 | 39,917001 | 41,184922 | 5 |
| 39,918840 | 41,184408 | 5 | 39,916873 | 41,184702 | 4 |
| 39,918598 | 41,187541 | 4 | 39,917129 | 41,184461 | 5 |
| 39,918359 | 41,187734 | 5 | 39,917277 | 41,184718 | 4 |
| 39,918125 | 41,187916 | 5 | 39,916507 | 41,183495 | 5 |
| 39,917874 | 41,188130 | 4 | 39,915553 | 41,183798 | 4 |
| 39,918479 | 41,187148 | 3 | 39,915446 | 41,184147 | 2 |
| 39,918249 | 41,187336 | 4 | 39,915215 | 41,183948 | 3 |
| 39,918068 | 41,187492 | 2 | 39,915326 | 41,183616 | 2 |
| 39,917883 | 41,187647 | 3 | 39,916101 | 41,189674 | 1 |
| 39,917714 | 41,187797 | 4 | 39,916365 | 41,189491 | 8 |
| 39,918253 | 41,186659 | 3 | 39,916579 | 41,189266 | 7 |
| 39,917994 | 41,186943 | 2 | 39,916809 | 41,189041 | 8 |
| 39,917706 | 41,187152 | 3 | 39,917052 | 41,188826 | 7 |
| 39,917484 | 41,187394 | 2 | 39,917266 | 41,188649 | 8 |
| 39,917356 | 41,187141 | 5 | 39,917463 | 41,188483 | 7 |
| 39,917912 | 41,186664 | 5 | 39,915929 | 41,189341 | 4 |
| 39,918134 | 41,186455 | 6 | 39,916130 | 41,189175 | 3 |

## Demand Points Coordinate and Demand (continued)

| **X Coordinate** | **Y Coordinate** | **Demand** | **X Coordinate** | **Y Coordinate** | **Demand** |
| --- | --- | --- | --- | --- | --- |
| 39,916303 | 41,189025 | 2 | 39,915670 | 41,185011 | 8 |
| 39,916451 | 41,188896 | 4 | 39,916099 | 41,186483 | 7 |
| 39,916591 | 41,188767 | 4 | 39,915976 | 41,186230 | 7 |
| 39,916764 | 41,188622 | 4 | 39,915840 | 41,185984 | 6 |
| 39,916924 | 41,188461 | 4 | 39,915737 | 41,185791 | 6 |
| 39,917114 | 41,188311 | 5 | 39,915511 | 41,185501 | 5 |
| 39,917299 | 41,188156 | 6 | 39,915259 | 41,184986 | 5 |
| 39,915725 | 41,188960 | 6 | 39,915033 | 41,184868 | 5 |
| 39,915918 | 41,188794 | 5 | 39,914807 | 41,184782 | 5 |
| 39,916108 | 41,188638 | 4 | 39,914597 | 41,184686 | 9 |
| 39,916285 | 41,188488 | 6 | 39,915099 | 41,185324 | 8 |
| 39,916601 | 41,188220 | 4 | 39,914869 | 41,185206 | 7 |
| 39,916420 | 41,187903 | 4 | 39,914630 | 41,185082 | 6 |
| 39,916239 | 41,188075 | 4 | 39,914292 | 41,185222 | 8 |
| 39,915927 | 41,188338 | 4 | 39,914086 | 41,185431 | 7 |
| 39,915750 | 41,188483 | 3 | 39,913897 | 41,185603 | 9 |
| 39,915565 | 41,188687 | 5 | 39,913695 | 41,185260 | 4 |
| 39,915336 | 41,187777 | 4 | 39,913888 | 41,185093 | 4 |
| 39,915989 | 41,187774 | 3 | 39,914090 | 41,184938 | 4 |
| 39,916248 | 41,187554 | 2 | 39,914304 | 41,184734 | 4 |
| 39,916137 | 41,187324 | 1 | 39,914843 | 41,185705 | 6 |
| 39,915870 | 41,187533 | 3 | 39,914588 | 41,185920 | 7 |
| 39,915742 | 41,187302 | 5 | 39,914345 | 41,186113 | 8 |
| 39,915998 | 41,187088 | 4 | 39,914213 | 41,186338 | 8 |
| 39,917075 | 41,187722 | 5 | 39,914061 | 41,186011 | 9 |
| 39,916828 | 41,187936 | 6 | 39,914217 | 41,185861 | 7 |
| 39,916697 | 41,187609 | 4 | 39,914386 | 41,185689 | 6 |
| 39,916804 | 41,187174 | 5 | 39,914592 | 41,185550 | 5 |
| 39,916581 | 41,187384 | 6 | 39,913977 | 41,184330 | 4 |
| 39,916437 | 41,187153 | 4 | 39,913767 | 41,184464 | 4 |
| 39,916697 | 41,186949 | 6 | 39,913627 | 41,184614 | 5 |
| 39,916339 | 41,186895 | 6 | 39,913421 | 41,184786 | 6 |
| 39,916615 | 41,186710 | 6 | 39,913249 | 41,184485 | 4 |
| 39,914917 | 41,187238 | 5 | 39,913454 | 41,184303 | 6 |
| 39,916423 | 41,186352 | 5 | 39,913640 | 41,184126 | 7 |
| 39,916304 | 41,186111 | 4 | 39,913257 | 41,183938 | 8 |
| 39,916201 | 41,185864 | 8 | 39,913277 | 41,183927 | 8 |
| 39,916114 | 41,185607 | 8 | 39,913084 | 41,183793 | 8 |
| 39,916028 | 41,185371 | 8 | 39,912854 | 41,184104 | 9 |
| 39,915818 | 41,185167 | 7 | 39,912858 | 41,183691 | 9 |

## Demand Points Coordinate and Demand (continued)

| **X Coordinate** | **Y Coordinate** | **Demand** | **X Coordinate** | **Y Coordinate** | **Demand** |
| --- | --- | --- | --- | --- | --- |
| 39,912615 | 41,183970 | 8 | 39,918950 | 41,195723 | 1 |
| 39,913158 | 41,185037 | 5 | 39,918752 | 41,195863 | 2 |
| 39,912985 | 41,185231 | 7 | 39,918589 | 41,195979 | 2 |
| 39,912837 | 41,185359 | 8 | 39,918709 | 41,196301 | 1 |
| 39,912644 | 41,185032 | 6 | 39,918906 | 41,196193 | 2 |
| 39,912068 | 41,184914 | 2 | 39,919092 | 41,196043 | 1 |
| 39,911990 | 41,183015 | 2 | 39,919289 | 41,195925 | 2 |
| 39,911833 | 41,183133 | 1 | 39,919445 | 41,195839 | 1 |
| 39,911426 | 41,183557 | 2 | 39,918395 | 41,196520 | 2 |
| 39,911574 | 41,183455 | 1 | 39,918214 | 41,196617 | 1 |
| 39,911290 | 41,183718 | 2 | 39,918037 | 41,196799 | 2 |
| 39,918852 | 41,202047 | 2 | 39,917814 | 41,196966 | 1 |
| 39,918901 | 41,201805 | 2 | 39,917679 | 41,196617 | 2 |
| 39,919012 | 41,201403 | 1 | 39,917901 | 41,196440 | 3 |
| 39,919086 | 41,201124 | 2 | 39,918127 | 41,196268 | 1 |
| 39,919136 | 41,200850 | 1 | 39,918321 | 41,196129 | 1 |
| 39,919226 | 41,200587 | 1 | 39,919693 | 41,196248 | 2 |
| 39,919284 | 41,200330 | 2 | 39,919841 | 41,196489 | 3 |
| 39,919358 | 41,200051 | 1 | 39,919886 | 41,196731 | 1 |
| 39,919436 | 41,199793 | 2 | 39,919821 | 41,196956 | 3 |
| 39,919499 | 41,199548 | 1 | 39,919771 | 41,197224 | 1 |
| 39,919587 | 41,199125 | 1 | 39,919467 | 41,197106 | 2 |
| 39,919661 | 41,198854 | 2 | 39,919467 | 41,197106 | 3 |
| 39,919735 | 41,198581 | 3 | 39,919533 | 41,196613 | 1 |
| 39,919793 | 41,198337 | 4 | 39,919426 | 41,196420 | 3 |
| 39,919870 | 41,198048 | 2 | 39,919170 | 41,197000 | 2 |
| 39,919960 | 41,197753 | 3 | 39,918981 | 41,197118 | 2 |
| 39,920081 | 41,197357 | 2 | 39,918648 | 41,197338 | 1 |
| 39,920139 | 41,197067 | 2 | 39,918382 | 41,197518 | 3 |
| 39,920200 | 41,196778 | 1 | 39,918139 | 41,197676 | 3 |
| 39,920283 | 41,195884 | 1 | 39,918234 | 41,197177 | 2 |
| 39,920147 | 41,195648 | 1 | 39,918505 | 41,196979 | 1 |
| 39,920011 | 41,195433 | 1 | 39,918830 | 41,196714 | 3 |
| 39,919900 | 41,195181 | 1 | 39,919069 | 41,196591 | 2 |
| 39,919583 | 41,195342 | 1 | 39,918579 | 41,198002 | 2 |
| 39,919719 | 41,195627 | 2 | 39,919651 | 41,197607 | 3 |
| 39,919871 | 41,195868 | 1 | 39,919564 | 41,197934 | 4 |
| 39,919999 | 41,196142 | 1 | 39,919503 | 41,198191 | 5 |
| 39,919299 | 41,195503 | 2 | 39,919441 | 41,198443 | 3 |
| 39,919159 | 41,195637 | 1 | 39,919363 | 41,198738 | 7 |

## Demand Points Coordinate and Demand (continued)

| **X Coordinate** | **Y Coordinate** | **Demand** | **X Coordinate** | **Y Coordinate** | **Demand** |
| --- | --- | --- | --- | --- | --- |
| 39,919285 | 41,198996 | 2 | 39,918239 | 41,200312 | 1 |
| 39,919174 | 41,199194 | 3 | 39,918046 | 41,200464 | 2 |
| 39,919013 | 41,199253 | 5 | 39,917115 | 41,202411 | 1 |
| 39,918836 | 41,199371 | 2 | 39,917538 | 41,202009 | 2 |
| 39,918680 | 41,198991 | 2 | 39,917418 | 41,201709 | 3 |
| 39,918902 | 41,198873 | 2 | 39,917159 | 41,201896 | 2 |
| 39,919079 | 41,198631 | 1 | 39,917238 | 41,201335 | 1 |
| 39,919157 | 41,198358 | 1 | 39,917053 | 41,201480 | 3 |
| 39,919239 | 41,198089 | 1 | 39,916846 | 41,201614 | 2 |
| 39,919293 | 41,197789 | 1 | 39,916677 | 41,201711 | 1 |
| 39,919363 | 41,197494 | 1 | 39,916549 | 41,201421 | 1 |
| 39,919474 | 41,197092 | 1 | 39,916718 | 41,201314 | 1 |
| 39,919519 | 41,196864 | 1 | 39,916928 | 41,201196 | 1 |
| 39,919523 | 41,196623 | 2 | 39,917117 | 41,201046 | 1 |
| 39,919424 | 41,196414 | 1 | 39,916932 | 41,200410 | 1 |
| 39,918675 | 41,201321 | 2 | 39,916833 | 41,200700 | 2 |
| 39,918462 | 41,201514 | 1 | 39,916624 | 41,200791 | 2 |
| 39,918149 | 41,201696 | 2 | 39,916414 | 41,200925 | 2 |
| 39,917886 | 41,201836 | 1 | 39,917387 | 41,199171 | 1 |
| 39,917803 | 41,201589 | 3 | 39,916341 | 41,201990 | 1 |
| 39,918046 | 41,201433 | 1 | 39,916205 | 41,201626 | 2 |
| 39,918322 | 41,201192 | 2 | 39,915966 | 41,201727 | 1 |
| 39,918503 | 41,201042 | 3 | 39,915835 | 41,201964 | 1 |
| 39,918696 | 41,200929 | 1 | 39,916036 | 41,201191 | 1 |
| 39,917680 | 41,201385 | 3 | 39,915773 | 41,201352 | 1 |
| 39,917947 | 41,201219 | 2 | 39,915522 | 41,201400 | 1 |
| 39,917577 | 41,201117 | 1 | 39,915263 | 41,201341 | 1 |
| 39,917470 | 41,200865 | 3 | 39,915213 | 41,200923 | 1 |
| 39,917853 | 41,200913 | 2 | 39,915469 | 41,200960 | 1 |
| 39,917754 | 41,200661 | 1 | 39,915703 | 41,200891 | 1 |
| 39,918153 | 41,200779 | 3 | 39,915901 | 41,200730 | 1 |
| 39,918355 | 41,200634 | 2 | 39,914526 | 41,201974 | 2 |
| 39,918536 | 41,200554 | 2 | 39,914662 | 41,201663 | 1 |
| 39,918741 | 41,200387 | 2 | 39,914358 | 41,201615 | 1 |
| 39,918955 | 41,200296 | 3 | 39,914242 | 41,201921 | 2 |
| 39,919169 | 41,199669 | 3 | 39,913707 | 41,201913 | 1 |
| 39,919005 | 41,199813 | 1 | 39,913768 | 41,201666 | 1 |
| 39,918811 | 41,199953 | 2 | 39,913888 | 41,201408 | 2 |
| 39,918622 | 41,200066 | 3 | 39,913390 | 41,201880 | 2 |
| 39,918433 | 41,200183 | 2 | 39,913488 | 41,201569 | 2 |

## Demand Points Coordinate and Demand (continued)

| **X Coordinate** | **Y Coordinate** | **Demand** | **X Coordinate** | **Y Coordinate** | **Demand** |
| --- | --- | --- | --- | --- | --- |
| 39,913583 | 41,201263 | 3 | 39,916883 | 41,197575 | 1 |
| 39,914032 | 41,201033 | 2 | 39,917167 | 41,197398 | 2 |
| 39,913789 | 41,200899 | 1 | 39,917426 | 41,197194 | 1 |
| 39,913567 | 41,200732 | 1 | 39,917290 | 41,196862 | 1 |
| 39,914151 | 41,200722 | 2 | 39,917010 | 41,197049 | 2 |
| 39,913970 | 41,200598 | 1 | 39,916782 | 41,197224 | 2 |
| 39,913776 | 41,200475 | 2 | 39,916486 | 41,197429 | 2 |
| 39,914706 | 41,200494 | 1 | 39,916222 | 41,197585 | 2 |
| 39,914768 | 41,200247 | 2 | 39,915976 | 41,197153 | 1 |
| 39,914842 | 41,200076 | 1 | 39,916642 | 41,196643 | 2 |
| 39,914295 | 41,200263 | 2 | 39,916815 | 41,196498 | 1 |
| 39,914398 | 41,199942 | 2 | 39,917037 | 41,196353 | 2 |
| 39,914505 | 41,199668 | 2 | 39,917193 | 41,196284 | 1 |
| 39,914616 | 41,199362 | 3 | 39,910331 | 41,201614 | 1 |
| 39,913995 | 41,200086 | 2 | 39,910558 | 41,201598 | 2 |
| 39,914110 | 41,199759 | 1 | 39,910768 | 41,201576 | 2 |
| 39,914196 | 41,199459 | 2 | 39,910566 | 41,201190 | 1 |
| 39,914283 | 41,199169 | 3 | 39,910903 | 41,201142 | 1 |
| 39,913624 | 41,199550 | 2 | 39,911035 | 41,200729 | 2 |
| 39,914768 | 41,198888 | 1 | 39,911212 | 41,201045 | 1 |
| 39,914538 | 41,198770 | 2 | 39,911364 | 41,201265 | 2 |
| 39,914571 | 41,198528 | 3 | 39,911496 | 41,201512 | 1 |
| 39,914937 | 41,198432 | 2 | 39,911632 | 41,201759 | 2 |
| 39,915031 | 41,197809 | 1 | 39,911932 | 41,201410 | 1 |
| 39,914834 | 41,197482 | 4 | 39,911833 | 41,201233 | 2 |
| 39,914620 | 41,197182 | 4 | 39,911743 | 41,201056 | 1 |
| 39,914340 | 41,197482 | 4 | 39,911623 | 41,200858 | 1 |
| 39,914604 | 41,197766 | 4 | 39,911525 | 41,200697 | 1 |
| 39,914879 | 41,198008 | 3 | 39,911430 | 41,200498 | 2 |
| 39,915530 | 41,199054 | 5 | 39,911327 | 41,200327 | 1 |
| 39,915666 | 41,198834 | 4 | 39,911554 | 41,200117 | 1 |
| 39,915834 | 41,198559 | 2 | 39,911673 | 41,200262 | 2 |
| 39,916019 | 41,198210 | 1 | 39,911759 | 41,200439 | 2 |
| 39,915866 | 41,197872 | 1 | 39,911858 | 41,200589 | 2 |
| 39,915591 | 41,198071 | 2 | 39,911944 | 41,200772 | 2 |
| 39,915434 | 41,198393 | 1 | 39,912039 | 41,200922 | 1 |
| 39,915311 | 41,198752 | 1 | 39,912134 | 41,201126 | 2 |
| 39,916261 | 41,198795 | 1 | 39,910893 | 41,199583 | 1 |
| 39,916364 | 41,197940 | 1 | 39,911139 | 41,199346 | 1 |
| 39,916624 | 41,197736 | 2 | 39,911341 | 41,199754 | 2 |

## Demand Points Coordinate and Demand (continued)

| **X Coordinate** | **Y Coordinate** | **Demand** | **X Coordinate** | **Y Coordinate** | **Demand** |
| --- | --- | --- | --- | --- | --- |
| 39,911111 | 41,199985 | 1 | 39,919296 | 41,193544 | 1 |
| 39,912754 | 41,200081 | 1 | 39,919230 | 41,193313 | 2 |
| 39,912750 | 41,199320 | 1 | 39,918889 | 41,193480 | 2 |
| 39,912333 | 41,198565 | 2 | 39,918998 | 41,193757 | 1 |
| 39,912193 | 41,198254 | 2 | 39,919060 | 41,193982 | 2 |
| 39,912028 | 41,197981 | 2 | 39,919200 | 41,194250 | 2 |
| 39,911864 | 41,197696 | 1 | 39,919315 | 41,194583 | 1 |
| 39,911625 | 41,197932 | 2 | 39,917500 | 41,196084 | 2 |
| 39,911790 | 41,198217 | 2 | 39,917767 | 41,195901 | 1 |
| 39,911938 | 41,198500 | 1 | 39,918063 | 41,195692 | 2 |
| 39,912107 | 41,198774 | 1 | 39,918396 | 41,195456 | 1 |
| 39,911599 | 41,197348 | 2 | 39,918656 | 41,195258 | 1 |
| 39,911500 | 41,197058 | 3 | 39,918902 | 41,195097 | 1 |
| 39,911377 | 41,197525 | 2 | 39,918804 | 41,194812 | 2 |
| 39,911282 | 41,197235 | 1 | 39,918565 | 41,194941 | 1 |
| 39,911081 | 41,197777 | 1 | 39,918269 | 41,195123 | 1 |
| 39,910845 | 41,197948 | 2 | 39,917940 | 41,195386 | 1 |
| 39,910940 | 41,197353 | 1 | 39,917652 | 41,195573 | 2 |
| 39,910717 | 41,197568 | 2 | 39,917379 | 41,195725 | 2 |
| 39,910804 | 41,196954 | 2 | 39,918033 | 41,194601 | 2 |
| 39,910610 | 41,197270 | 1 | 39,918827 | 41,194236 | 3 |
| 39,910532 | 41,198268 | 4 | 39,918749 | 41,194000 | 4 |
| 39,910421 | 41,197839 | 4 | 39,918642 | 41,193753 | 3 |
| 39,910261 | 41,198391 | 4 | 39,918358 | 41,193962 | 1 |
| 39,910195 | 41,198150 | 2 | 39,918449 | 41,194182 | 1 |
| 39,910404 | 41,196211 | 1 | 39,918544 | 41,194440 | 2 |
| 39,910111 | 41,196308 | 1 | 39,918441 | 41,193309 | 3 |
| 39,909869 | 41,196093 | 1 | 39,918276 | 41,192912 | 1 |
| 39,908865 | 41,195677 | 2 | 39,917865 | 41,194022 | 2 |
| 39,908333 | 41,195935 | 1 | 39,917564 | 41,193904 | 2 |
| 39,908460 | 41,196326 | 2 | 39,917420 | 41,194113 | 3 |
| 39,908053 | 41,196122 | 1 | 39,917276 | 41,194387 | 2 |
| 39,908201 | 41,196509 | 3 | 39,917745 | 41,189056 | 4 |
| 39,907790 | 41,196315 | 2 | 39,917543 | 41,189240 | 2 |
| 39,907917 | 41,196702 | 2 | 39,917300 | 41,189444 | 2 |
| 39,907666 | 41,196830 | 2 | 39,917069 | 41,189642 | 2 |
| 39,907543 | 41,196487 | 2 | 39,916838 | 41,189869 | 1 |
| 39,919588 | 41,194419 | 2 | 39,916616 | 41,190048 | 2 |
| 39,919481 | 41,194091 | 1 | 39,916410 | 41,190246 | 3 |
| 39,919395 | 41,193807 | 2 | 39,916552 | 41,190593 | 4 |

## Demand Points Coordinate and Demand (continued)

| **X Coordinate** | **Y Coordinate** | **Demand** | **X Coordinate** | **Y Coordinate** | **Demand** |
| --- | --- | --- | --- | --- | --- |
| 39,916757 | 41,190432 | 3 | 39,914002 | 41,192344 | 5 |
| 39,916926 | 41,190298 | 5 | 39,913755 | 41,192542 | 6 |
| 39,917111 | 41,190137 | 4 | 39,913492 | 41,192682 | 6 |
| 39,917288 | 41,189960 | 2 | 39,913295 | 41,192875 | 7 |
| 39,918001 | 41,189928 | 3 | 39,912933 | 41,193202 | 7 |
| 39,918121 | 41,190212 | 2 | 39,912686 | 41,193400 | 7 |
| 39,918199 | 41,190438 | 3 | 39,916237 | 41,190856 | 8 |
| 39,918269 | 41,190700 | 3 | 39,916039 | 41,191033 | 6 |
| 39,918417 | 41,191054 | 4 | 39,915838 | 41,191242 | 5 |
| 39,917779 | 41,190175 | 4 | 39,915648 | 41,191377 | 4 |
| 39,917875 | 41,190393 | 4 | 39,915467 | 41,191543 | 4 |
| 39,917958 | 41,190666 | 5 | 39,915134 | 41,191847 | 3 |
| 39,918027 | 41,190913 | 3 | 39,914928 | 41,192003 | 3 |
| 39,918237 | 41,191534 | 3 | 39,914743 | 41,192174 | 2 |
| 39,918320 | 41,191777 | 3 | 39,914529 | 41,192335 | 2 |
| 39,917505 | 41,190328 | 3 | 39,914176 | 41,192624 | 1 |
| 39,917345 | 41,190484 | 4 | 39,913987 | 41,192763 | 2 |
| 39,917147 | 41,190618 | 3 | 39,913790 | 41,192951 | 3 |
| 39,916971 | 41,190784 | 3 | 39,913617 | 41,193079 | 2 |
| 39,916740 | 41,190977 | 4 | 39,913427 | 41,193235 | 2 |
| 39,916942 | 41,191278 | 3 | 39,913127 | 41,193466 | 3 |
| 39,917115 | 41,191117 | 5 | 39,912851 | 41,193686 | 2 |
| 39,917337 | 41,190940 | 4 | 39,916439 | 41,191264 | 1 |
| 39,917542 | 41,190741 | 2 | 39,916258 | 41,191419 | 3 |
| 39,917296 | 41,191967 | 3 | 39,916069 | 41,191596 | 2 |
| 39,917115 | 41,191650 | 5 | 39,915909 | 41,191741 | 1 |
| 39,917308 | 41,191489 | 2 | 39,915727 | 41,191902 | 3 |
| 39,917460 | 41,191827 | 4 | 39,916616 | 41,191586 | 2 |
| 39,917633 | 41,191682 | 4 | 39,916415 | 41,191747 | 2 |
| 39,917489 | 41,191328 | 4 | 39,916234 | 41,191886 | 2 |
| 39,917674 | 41,191183 | 4 | 39,916070 | 41,192056 | 2 |
| 39,917818 | 41,191511 | 3 | 39,915877 | 41,192207 | 2 |
| 39,917761 | 41,192305 | 3 | 39,915428 | 41,192160 | 2 |
| 39,916048 | 41,190601 | 2 | 39,915292 | 41,192299 | 1 |
| 39,915768 | 41,190810 | 5 | 39,915107 | 41,192439 | 3 |
| 39,915554 | 41,191008 | 7 | 39,914926 | 41,192578 | 3 |
| 39,915311 | 41,191218 | 7 | 39,914737 | 41,192761 | 2 |
| 39,914908 | 41,191582 | 7 | 39,915622 | 41,192444 | 2 |
| 39,914637 | 41,191786 | 5 | 39,915440 | 41,192589 | 4 |
| 39,914378 | 41,192011 | 5 | 39,915264 | 41,192745 | 5 |

## Demand Points Coordinate and Demand (continued)

| **X Coordinate** | **Y Coordinate** | **Demand** | **X Coordinate** | **Y Coordinate** | **Demand** |
| --- | --- | --- | --- | --- | --- |
| 39,915091 | 41,192911 | 4 | 39,915586 | 41,193095 | 4 |
| 39,914901 | 41,193050 | 5 | 39,915713 | 41,193326 | 3 |
| 39,914411 | 41,193030 | 6 | 39,915866 | 41,193573 | 5 |
| 39,914201 | 41,193186 | 4 | 39,915996 | 41,193820 | 4 |
| 39,914028 | 41,193336 | 3 | 39,916166 | 41,194054 | 3 |
| 39,913843 | 41,193519 | 2 | 39,915256 | 41,193303 | 2 |
| 39,913625 | 41,193615 | 4 | 39,915512 | 41,193716 | 1 |
| 39,914612 | 41,193293 | 3 | 39,915228 | 41,193904 | 3 |
| 39,914452 | 41,193422 | 2 | 39,914960 | 41,193582 | 4 |
| 39,914238 | 41,193588 | 2 | 39,914668 | 41,193770 | 5 |
| 39,914065 | 41,193728 | 2 | 39,915565 | 41,194558 | 3 |
| 39,913855 | 41,193883 | 4 | 39,914377 | 41,194048 | 2 |
| 39,913257 | 41,193712 | 3 | 39,914517 | 41,194278 | 1 |
| 39,913022 | 41,193905 | 4 | 39,914665 | 41,194504 | 5 |
| 39,913409 | 41,193916 | 2 | 39,914821 | 41,194740 | 3 |
| 39,913125 | 41,194114 | 3 | 39,914587 | 41,194922 | 4 |
| 39,913426 | 41,193910 | 4 | 39,914410 | 41,194707 | 3 |
| 39,913232 | 41,194296 | 3 | 39,914274 | 41,194493 | 3 |
| 39,913557 | 41,194162 | 3 | 39,914097 | 41,194262 | 5 |
| 39,913364 | 41,194420 | 2 | 39,913763 | 41,194477 | 4 |
| 39,917475 | 41,193294 | 1 | 39,913919 | 41,194659 | 3 |
| 39,917331 | 41,193503 | 4 | 39,914088 | 41,194906 | 2 |
| 39,917183 | 41,193718 | 3 | 39,914211 | 41,195115 | 3 |
| 39,917022 | 41,193948 | 3 | 39,914051 | 41,195421 | 1 |
| 39,916837 | 41,193643 | 3 | 39,913894 | 41,195233 | 3 |
| 39,916985 | 41,193412 | 4 | 39,913734 | 41,194992 | 5 |
| 39,917146 | 41,193224 | 3 | 39,913586 | 41,194782 | 1 |
| 39,917319 | 41,192972 | 4 | 39,915462 | 41,197556 | 3 |
| 39,916783 | 41,193026 | 4 | 39,915330 | 41,197363 | 4 |
| 39,916639 | 41,192816 | 3 | 39,915211 | 41,197154 | 3 |
| 39,916499 | 41,192554 | 2 | 39,915051 | 41,196966 | 2 |
| 39,916347 | 41,192301 | 2 | 39,914878 | 41,196762 | 3 |
| 39,916133 | 41,192564 | 3 | 39,914738 | 41,196596 | 2 |
| 39,916306 | 41,192790 | 3 | 39,914602 | 41,196349 | 1 |
| 39,916442 | 41,193069 | 4 | 39,914450 | 41,196129 | 2 |
| 39,916582 | 41,193272 | 3 | 39,914302 | 41,195893 | 3 |
| 39,915796 | 41,192859 | 2 | 39,915680 | 41,197314 | 2 |
| 39,915944 | 41,193117 | 2 | 39,915565 | 41,197132 | 1 |
| 39,916080 | 41,193353 | 4 | 39,915495 | 41,196837 | 2 |
| 39,916232 | 41,193600 | 4 | 39,915318 | 41,196671 | 3 |

## Demand Points Coordinate and Demand (continued)

| **X Coordinate** | **Y Coordinate** | **Demand** | **X Coordinate** | **Y Coordinate** | **Demand** |
| --- | --- | --- | --- | --- | --- |
| 39,915207 | 41,196429 | 2 | 39,915244 | 41,190415 | 7 |
| 39,915042 | 41,196188 | 1 | 39,914973 | 41,190684 | 8 |
| 39,914903 | 41,196011 | 4 | 39,915611 | 41,189670 | 9 |
| 39,914755 | 41,195791 | 4 | 39,915413 | 41,189847 | 9 |
| 39,914602 | 41,195582 | 3 | 39,915199 | 41,190010 | 8 |
| 39,914907 | 41,195399 | 4 | 39,915002 | 41,190208 | 6 |
| 39,915146 | 41,195137 | 4 | 39,914804 | 41,190342 | 8 |
| 39,915051 | 41,195635 | 3 | 39,914561 | 41,191060 | 4 |
| 39,915294 | 41,195389 | 4 | 39,914318 | 41,191248 | 8 |
| 39,915224 | 41,195871 | 3 | 39,914100 | 41,191430 | 7 |
| 39,915339 | 41,196075 | 4 | 39,914475 | 41,190625 | 6 |
| 39,915438 | 41,195598 | 3 | 39,914228 | 41,190781 | 8 |
| 39,915578 | 41,195818 | 4 | 39,914084 | 41,190931 | 7 |
| 39,915713 | 41,196027 | 4 | 39,913882 | 41,191103 | 6 |
| 39,915837 | 41,196188 | 4 | 39,913717 | 41,191768 | 7 |
| 39,917242 | 41,194990 | 4 | 39,913471 | 41,191950 | 8 |
| 39,917094 | 41,195259 | 4 | 39,913236 | 41,192154 | 7 |
| 39,916991 | 41,194787 | 5 | 39,912997 | 41,192342 | 8 |
| 39,916855 | 41,195012 | 4 | 39,912845 | 41,191961 | 8 |
| 39,916732 | 41,195253 | 3 | 39,912442 | 41,192658 | 8 |
| 39,916621 | 41,195473 | 5 | 39,913532 | 41,191392 | 8 |
| 39,916489 | 41,195698 | 4 | 39,913294 | 41,191586 | 2 |
| 39,916337 | 41,195977 | 2 | 39,915404 | 41,189273 | 3 |
| 39,916835 | 41,195666 | 1 | 39,915207 | 41,189482 | 4 |
| 39,916738 | 41,194314 | 1 | 39,914993 | 41,189600 | 5 |
| 39,916611 | 41,194567 | 2 | 39,914820 | 41,189788 | 3 |
| 39,916496 | 41,194781 | 2 | 39,914594 | 41,189922 | 5 |
| 39,916393 | 41,195049 | 1 | 39,915244 | 41,188951 | 4 |
| 39,916257 | 41,195275 | 3 | 39,915030 | 41,189123 | 6 |
| 39,916175 | 41,195521 | 2 | 39,914849 | 41,189252 | 6 |
| 39,916031 | 41,195763 | 1 | 39,914672 | 41,189450 | 5 |
| 39,916533 | 41,194121 | 3 | 39,914450 | 41,189676 | 5 |
| 39,916409 | 41,194347 | 2 | 39,914262 | 41,190226 | 5 |
| 39,916278 | 41,194561 | 2 | 39,914027 | 41,190403 | 4 |
| 39,916167 | 41,194808 | 2 | 39,913789 | 41,190607 | 4 |
| 39,916006 | 41,195028 | 2 | 39,914114 | 41,189958 | 4 |
| 39,915874 | 41,195270 | 2 | 39,913883 | 41,190113 | 4 |
| 39,915750 | 41,195544 | 4 | 39,913673 | 41,190296 | 2 |
| 39,915759 | 41,190018 | 5 | 39,913509 | 41,190425 | 3 |
| 39,915487 | 41,190206 | 6 | 39,913357 | 41,191020 | 5 |

## Demand Points Coordinate and Demand (continued)

| **X Coordinate** | **Y Coordinate** | **Demand** | **X Coordinate** | **Y Coordinate** | **Demand** |
| --- | --- | --- | --- | --- | --- |
| 39,913204 | 41,190666 | 4 | 39,913642 | 41,187369 | 3 |
| 39,913126 | 41,191186 | 3 | 39,913795 | 41,187605 | 3 |
| 39,912986 | 41,190827 | 8 | 39,913900 | 41,187911 | 2 |
| 39,912912 | 41,191353 | 6 | 39,913624 | 41,188035 | 8 |
| 39,912776 | 41,190966 | 4 | 39,913460 | 41,185549 | 9 |
| 39,912707 | 41,191481 | 8 | 39,913645 | 41,185844 | 4 |
| 39,912587 | 41,191127 | 8 | 39,913279 | 41,185699 | 4 |
| 39,912128 | 41,191998 | 7 | 39,913480 | 41,185989 | 4 |
| 39,912062 | 41,191761 | 6 | 39,913112 | 41,185905 | 4 |
| 39,911967 | 41,191450 | 5 | 39,913289 | 41,186200 | 4 |
| 39,911893 | 41,191230 | 4 | 39,912923 | 41,186039 | 4 |
| 39,911803 | 41,190968 | 6 | 39,913026 | 41,186420 | 5 |
| 39,912379 | 41,191713 | 5 | 39,912717 | 41,186248 | 4 |
| 39,912301 | 41,191466 | 7 | 39,912886 | 41,186570 | 5 |
| 39,912214 | 41,191193 | 6 | 39,912481 | 41,186437 | 6 |
| 39,912128 | 41,190935 | 8 | 39,912279 | 41,186598 | 3 |
| 39,912440 | 41,190729 | 7 | 39,912078 | 41,186721 | 5 |
| 39,912350 | 41,190461 | 3 | 39,911876 | 41,186898 | 2 |
| 39,912671 | 41,190504 | 5 | 39,911691 | 41,187054 | 7 |
| 39,912613 | 41,190284 | 7 | 39,911847 | 41,187440 | 7 |
| 39,912905 | 41,189495 | 8 | 39,912238 | 41,187091 | 7 |
| 39,914512 | 41,189063 | 6 | 39,912456 | 41,186936 | 8 |
| 39,914265 | 41,189235 | 7 | 39,912641 | 41,186791 | 8 |
| 39,914364 | 41,188795 | 8 | 39,912785 | 41,187177 | 6 |
| 39,914134 | 41,188999 | 2 | 39,912534 | 41,187413 | 6 |
| 39,914241 | 41,188564 | 3 | 39,912892 | 41,187445 | 6 |
| 39,914002 | 41,188773 | 3 | 39,912641 | 41,187670 | 6 |
| 39,914134 | 41,188258 | 4 | 39,913024 | 41,187719 | 6 |
| 39,913886 | 41,188487 | 5 | 39,912794 | 41,187923 | 8 |
| 39,913935 | 41,189512 | 6 | 39,913168 | 41,187987 | 6 |
| 39,913795 | 41,189233 | 5 | 39,912905 | 41,188202 | 6 |
| 39,913474 | 41,189308 | 4 | 39,913312 | 41,188303 | 8 |
| 39,913598 | 41,189592 | 4 | 39,913032 | 41,188480 | 7 |
| 39,913680 | 41,189769 | 6 | 39,911370 | 41,189141 | 6 |
| 39,914350 | 41,186968 | 5 | 39,911139 | 41,189313 | 6 |
| 39,913980 | 41,186528 | 3 | 39,911275 | 41,188857 | 6 |
| 39,913840 | 41,186265 | 2 | 39,911016 | 41,189098 | 5 |
| 39,913659 | 41,186425 | 4 | 39,911123 | 41,188637 | 4 |
| 39,913741 | 41,186747 | 8 | 39,910909 | 41,188814 | 4 |
| 39,913490 | 41,186586 | 4 | 39,911524 | 41,187705 | 4 |

## Demand Points Coordinate and Demand (continued)

| **X Coordinate** | **Y Coordinate** | **Demand** | **X Coordinate** | **Y Coordinate** | **Demand** |
| --- | --- | --- | --- | --- | --- |
| 39,911322 | 41,187882 | 4 | 39,909972 | 41,184861 | 4 |
| 39,911137 | 41,187989 | 5 | 39,910158 | 41,184668 | 3 |
| 39,910956 | 41,188183 | 5 | 39,910121 | 41,188326 | 2 |
| 39,910779 | 41,188301 | 4 | 39,909895 | 41,188540 | 4 |
| 39,910598 | 41,187920 | 4 | 39,910298 | 41,188664 | 2 |
| 39,910779 | 41,187769 | 4 | 39,909825 | 41,189088 | 3 |
| 39,910972 | 41,187651 | 8 | 39,909442 | 41,189538 | 2 |
| 39,911396 | 41,187356 | 8 | 39,909689 | 41,189882 | 1 |
| 39,910201 | 41,187533 | 8 | 39,909907 | 41,189651 | 2 |
| 39,910387 | 41,187410 | 7 | 39,910195 | 41,189356 | 5 |
| 39,910539 | 41,187254 | 6 | 39,910376 | 41,189200 | 4 |
| 39,910724 | 41,187099 | 5 | 39,910940 | 41,189999 | 7 |
| 39,910909 | 41,186976 | 5 | 39,910653 | 41,190237 | 5 |
| 39,911090 | 41,186853 | 8 | 39,910246 | 41,190254 | 4 |
| 39,911345 | 41,186617 | 7 | 39,910377 | 41,190511 | 8 |
| 39,911535 | 41,186505 | 7 | 39,910003 | 41,190474 | 7 |
| 39,911526 | 41,186043 | 9 | 39,910188 | 41,190736 | 6 |
| 39,910926 | 41,186515 | 8 | 39,907902 | 41,189240 | 5 |
| 39,910728 | 41,186671 | 6 | 39,908030 | 41,189573 | 5 |
| 39,910551 | 41,186789 | 4 | 39,908281 | 41,189407 | 4 |
| 39,910374 | 41,186928 | 6 | 39,907166 | 41,189187 | 3 |
| 39,910193 | 41,187057 | 8 | 39,907318 | 41,189637 | 2 |
| 39,910033 | 41,187197 | 7 | 39,908293 | 41,190581 | 2 |
| 39,909932 | 41,186805 | 5 | 39,908273 | 41,189986 | 3 |
| 39,910187 | 41,186644 | 5 | 39,908030 | 41,191472 | 2 |
| 39,910089 | 41,186354 | 6 | 39,908124 | 41,191770 | 1 |
| 39,909838 | 41,186580 | 6 | 39,910873 | 41,193233 | 2 |
| 39,909736 | 41,186283 | 4 | 39,910400 | 41,193549 | 3 |
| 39,909913 | 41,185880 | 4 | 39,910301 | 41,194107 | 2 |
| 39,909658 | 41,186057 | 2 | 39,910107 | 41,193678 | 2 |
| 39,909617 | 41,187055 | 6 | 39,909898 | 41,194274 | 1 |
| 39,909518 | 41,186851 | 4 | 39,909692 | 41,194376 | 2 |
| 39,909300 | 41,187050 | 2 | 39,908622 | 41,194622 | 1 |
| 39,909284 | 41,186508 | 3 | 39,909161 | 41,194225 | 3 |
| 39,908971 | 41,186723 | 4 | 39,909050 | 41,193785 | 2 |
| 39,908782 | 41,186942 | 5 |  |  |  |
| 39,908543 | 41,185993 | 2 |  |  |  |
| 39,909376 | 41,185328 | 2 |  |  |  |
| 39,909586 | 41,185151 | 1 |  |  |  |
| 39,909754 | 41,185022 | 3 |  |  |  |

## Station Coordinate

| **Station X Coordinate** | **Station Y Coordinate** |
| --- | --- |
| 39,922258 | 41,187756 |
| 39,921836 | 41,188689 |
| 39,920539 | 41,188357 |
| 39,919953 | 41,189032 |
| 39,922325 | 41,189987 |
| 39,920691 | 41,191368 |
| 39,920683 | 41,193610 |
| 39,918519 | 41,191836 |
| 39,919080 | 41,190892 |
| 39,917889 | 41,192196 |
| 39,916890 | 41,192196 |
| 39,918244 | 41,194329 |
| 39,916700 | 41,198514 |
| 39,917352 | 41,199052 |
| 39,918476 | 41,201809 |
| 39,916296 | 41,198670 |
| 39,915500 | 41,194365 |
| 39,919130 | 41,185866 |
| 39,918015 | 41,184535 |
| 39,916503 | 41,183614 |
| 39,914799 | 41,187228 |
| 39,914363 | 41,186880 |
| 39,915311 | 41,185622 |
| 39,910938 | 41,185659 |
| 39,915417 | 41,187851 |
| 39,913385 | 41,189791 |
| 39,912619 | 41,192256 |
| 39,911825 | 41,193179 |
| 39,912826 | 41,194336 |
| 39,913744 | 41,195666 |
| 39,911286 | 41,198756 |
| 39,912201 | 41,188007 |
| 39,912701 | 41,188737 |
| 39,912784 | 41,189252 |
| 39,917716 | 41,185788 |
| 39,920829 | 41,195184 |
| 39,918868 | 41,200634 |
| 39,919424 | 41,188671 |
| 39,911693 | 41,195890 |
| 39,912372 | 41,183895 |
